# Next-generation smart polymeric hydrogels for chronic wound management: integrating antimicrobial, immunomodulatory and regenerative strategies

**DOI:** 10.1039/d6ra06982g

**Published:** 2026-10-05

**Authors:** Muhammad Bilal Habib, Laiba Arshad, Muhammad Kamran, Ghanwa Batool, Taseer Muhammad, Anwarul Hasan, Noreen Sher Akbar, Ameera Shahid

**Affiliations:** a Department of Biosciences, Comsats University Islamabad 45550 Pakistan b.habib@nih.org.pk; b National University of Medical Sciences Islamabad 45550 Pakistan arshadlaiba679@gmail.com; c Department of Allied Health Sciences, Government College Women University Faisalabad Punjab Pakistan muhammadkamran@gcwuf.edu.pk; d Department of Computer Science, Comsats University Abbottabad Campus 22060 Pakistan ghanwa@cuiatd.edu.pk; e Department of Mathematics, College of Science, King Khalid University Abha 61413 Saudi Arabia tasgher@kku.edu.sa; f Department of Bioengineering, King Fahd University of Petroleum and Minerals (KFUPM) Dhahran 31261 Saudi Arabia mdanwarul.hasan@kfupm.edu.sa; g Department of Mechanical Engineering, College of Engineering, Prince Mohammad Bin Fahd University Al Khobar 31952 Saudi Arabia nakbar@pmu.edu.sa; h College of Medical Laboratory Technology, National Institute of Health Islamabad 45500 Pakistan ameerashahid6@gmail.com

## Abstract

Wound healing is a tightly orchestrated sequence of hemostatic, inflammatory, proliferative, and remodeling events, yet chronic wounds arise when this cascade is dysregulated by persistent infection, excessive oxidative stress, and biofilm formation. Such pathological conditions particularly in diabetic and vascular-compromised patients sustain inflammatory signaling, impair angiogenesis, and disrupt extracellular matrix (ECM) turnover. In this study, we comprehensively evaluated these pathological barriers while presenting an integrated overview of recent advances in multifunctional smart hydrogels engineered to overcome them. Rather than treating antimicrobial, immunomodulatory and regenerative functions as independent therapeutic categories, this review established a microenvironment material function translation framework emphasizing their temporal and spatial coordination. We further critically examined dynamic network chemistries, nanocomposite and multicomponent architectures, advanced 3D/4D fabrication, AI-assisted formulation and wound monitoring, preclinical model limitations, manufacturing scalability and regulatory barriers. By distinguishing promising proof-of-concept strategies from technologies approaching clinical translation, this review identifies design principles for safer, more reproducible and clinically relevant smart hydrogel systems. Finally, we addressed translational barriers including scalability, regulatory classification, and long-term biocompatibility and outline future directions for AI-integrated, patient-specific hydrogel systems. Collectively, this study provides a comprehensive technical assessment of next-generation polymeric hydrogels as precision platforms for advanced wound management.

## Abstract 2

Multifunctional hydrogel for wound healing, showing the polymer network, embedded antibacterial agents, stimuli-responsive crosslinks, and bioactive drug release.

## Graphical abstract

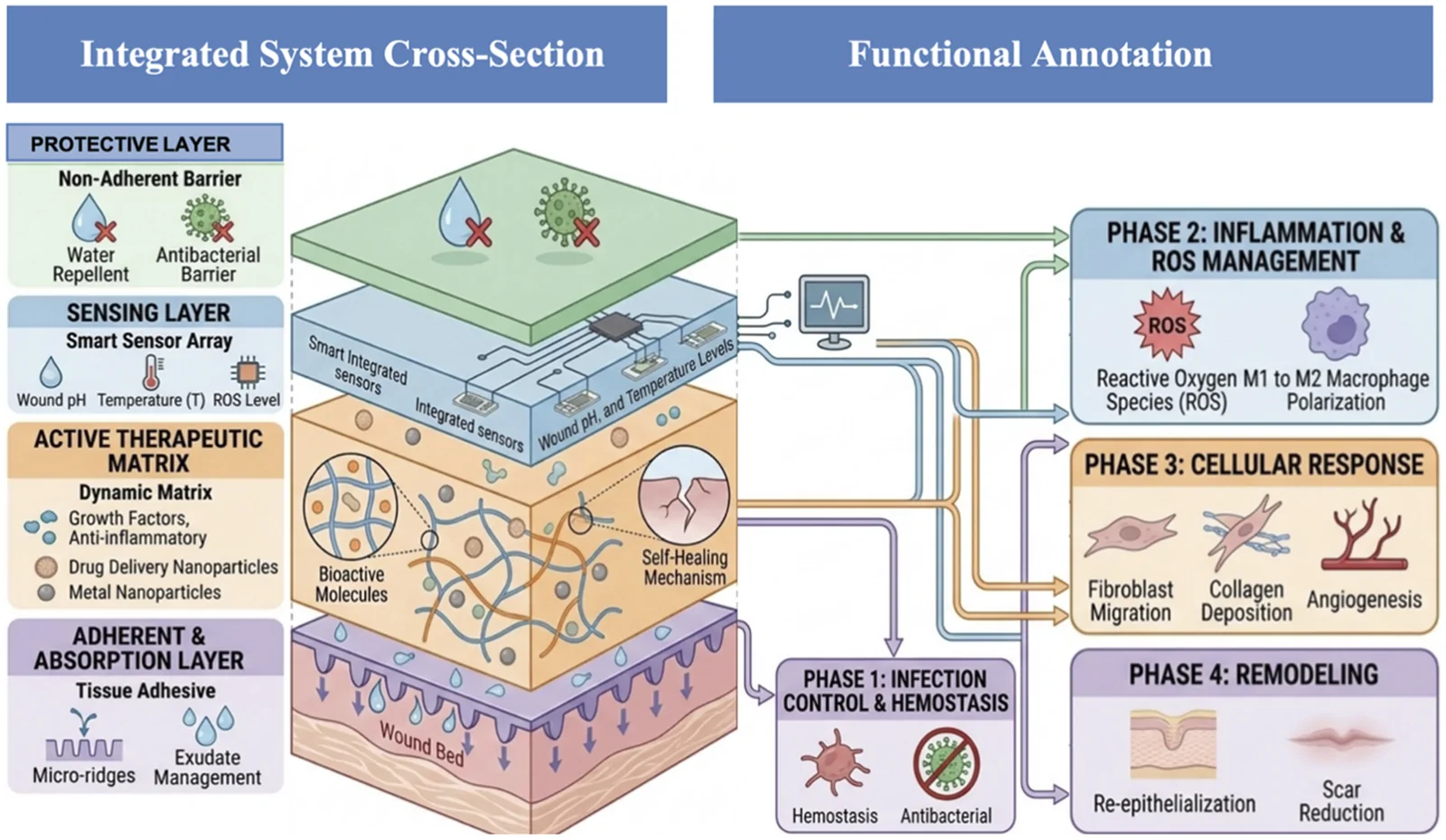

## 1. Introduction

### 1.1. Overview of wound healing challenges

Wound healing is a complex biological process that restores the integrity of injured tissue. Under optimal conditions, healing proceeds through a well-coordinated sequence of phases: haemostasias, inflammation, proliferation (granulation, angiogenesis, re-epithelialization) and remodeling (maturation).^1^ In contrast, when one or more of these phases is disrupted, healing may be delayed or fail altogether, leading to chronic wounds. Under normal physiological conditions, acute wounds (such as surgical incisions or trauma) often heal in a matter of days to weeks. On the other hand, chronic wounds present a major therapeutic challenge since they remain in one or more stages (often the inflammatory or proliferative stage) for prolonged periods of time, lasting from weeks to months.^2,3^ For instance, diabetic foot ulcers, pressure ulcers, and venous leg ulcers are frequently categorized as chronic diseases. Due to neuropathy (loss of feeling), ischemia (microvascular problems), and a compromised immune system, patients with diabetes mellitus are more likely to get foot ulcers. These ulcers are made worse by persistent inflammation, elevated reactive oxygen species, increased matrix metalloproteinases (MMPs) activity, inadequate angiogenesis, and a significant risk of infection or amputation if left untreated.^4^

One major obstacle to wound healing is microbial colonization, especially the development of biofilms on the implants or wound bed. By extending the inflammatory phase, breaking down the extracellular matrix (ECM), and postponing re-epithelialization, biofilms obstruct healing and shield germs from immune destruction and therapies. Implant-related infections are particularly important because they make foreign-body responses and biofilm formation on biomaterials more difficult.^5^ Exudate management, moisture equilibrium maintenance, mechanical pressure on the wound site, pre-existing medical disorders including vascular insufficiency, and patient characteristics like age, nutritional status, and mobility all have an impact on healing outcomes. An unfavorable microenvironment that is marked by an excess of exudate, the presence of germs, restricted cellular mobility, decreased growth factor efficiency, and poor angiogenesis is fostered by chronic wounds. The wound microenvironment's multi-phase architecture should be recognized by hydrogels.^6^ The distinction between acute and chronic wound healing is shown in Scheme 1. Wound dressings and treatment approaches should go beyond basic covering in light of these consequences. They must actively control the wound environment, promote angiogenesis and extracellular matrix reorganization, facilitate cell migration, and manage infection.

**Scheme 1:**
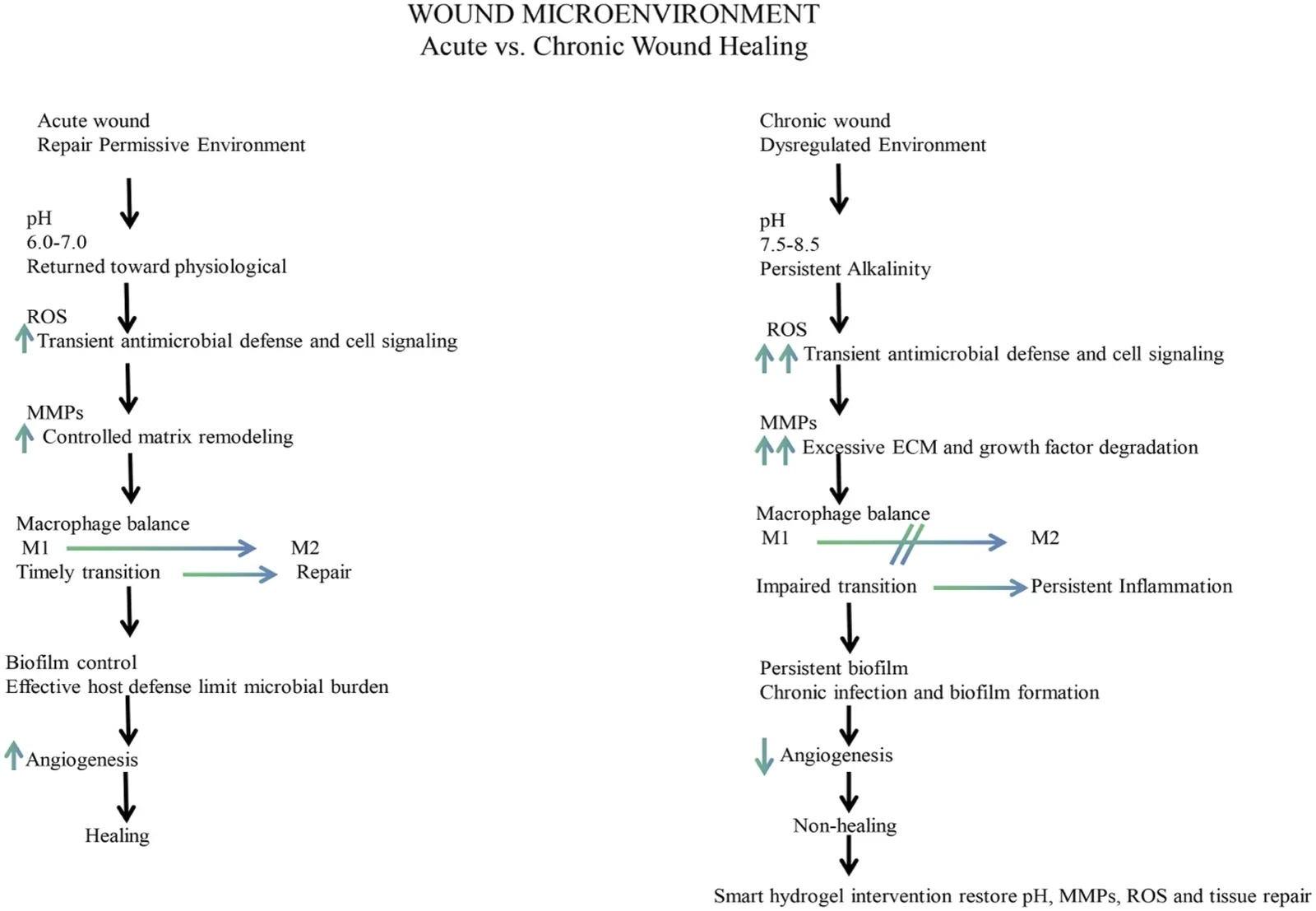
Schematic illustration of the mechanisms and effects of pH, ROS, MMPs, macrophage balance, biofilm control in acute *vs.* chronic wounds and intervention of smart hydrogels for recovery of chronic wounds.

### 1.2. Importance of multifunctional hydrogel systems in modern wound care

In wound care, hydrogels three-dimensional, hydrophilic polymer networks with a high water retention capacity have become important biomaterials. They can conform to uneven wound beds, absorb exudate, create a moist environment, and be designed to release bioactive substances. Recent developments, however, concentrate on multifunctional hydrogels, which combine multiple therapeutic purposes into a single solution. The review, “Rational Design of Multifunctional Hydrogels for Wound Repair” stated that multifunctional hydrogels meet a number of wound-care needs, including the maintenance of a moist environment, adhesion to wound surfaces, regulation of permeation (oxygen, nutrients, waste removal), and active therapeutic roles like immunomodulatory, pro-angiogenic, antibacterial, and antioxidant effects.^7^ Hydrogels that provide anti-inflammatory, antibacterial, immunomodulatory, and angiogenic stimuli specific to the diabetic wound conditions are also emphasized in recent research on diabetic wound repair. Biofilm generation and bacterial burden can be avoided by including antimicrobial chemicals, nanoparticles, or bacteriostatic surfaces.^8^

Since chronic wounds frequently remains in the inflammatory stage, hydrogels may include anti-inflammatory medicines, reactive oxygen species scavengers, or matrix metalloproteinase inhibitors to change the healing process. Growth factors, cells, or angiogenic signals added to hydrogels promote the proliferative phase, which is marked by granulation tissue development and revascularization.^9,10^ From flexible conductive materials, hydrogels have developed into all-encompassing facilitators that connect biology and electronics, enabling integrated sensing, modulation, and adaptive control in physiological systems. By integrating sensing, stimulation, and response behaviors inside a hydrated and biocompatible matrix, hydrogel-based technologies are transforming physiological monitoring and therapeutic modulation in the brain, gastrointestinal, and cardiac domains.^11^ Hydrogels were mostly utilized as passive wound dressing coverings in the past. They absorb exudate, maintain moisture, provide a soft, conformable contact, and protect the area from external pollutants. Collagen, alginate, chitosan, and crosslinked polyacrylamide are examples of simple hydrogels that passively transmit moisture and regulate exudate. These solutions are advantageous over dry dressings, but their capacity to actively react to the intricate wound microenvironment (risk of infection, pH/ROS fluctuations, impeded angiogenesis, *etc.*) is constrained.^12^ Smart or stimuli-responsive hydrogels, on the other hand, are designed to react to internal or external stimuli (such as pH, temperature, reactive oxygen species, enzymes, light, and magnetic fields) in order to produce on-demand behaviors, such as precise adhesion, controlled drug release, shape-change/adaptive swelling, and activation of antimicrobial activity. For instance, light-activated hydrogels can gel *in situ* or initiate antibacterial action when exposed to near-infrared radiation; hydrogels that react to high ROS in a chronic wound can release antioxidant compounds or disintegrate to release therapeutic payloads.^13^ The main characteristics that set smart hydrogel systems apart from conventional ones are listed in Table 1.

**Table 1:** Comparison of traditional *vs.* smart hydrogel wound-dressings

Traditional hydrogel dressings	Smart responsive hydrogel systems	References
Moisture retention, exudate absorption and passive protection	Active modulation of wound micro-environment including infection, inflammation and angiogenesis	14
Limited or no responsiveness to changes in the wound environment	Responsive to stimuli like pH, ROS, light, enzyme and temperature	15
Mostly passive drug release, with limited control over therapeutic availability	Controlled, on-demand or triggered delivery	16
Generally, provide a generic wound environment	Designed for specific wound types *e.g.*, diabetic, infected, implant-associated	17
Relatively simple fabrication and lower cost	More complex fabrication, higher cost, stronger design demands	18

From a materials perspective, smart hydrogels often incorporate functional moieties (*e.g.*, stimuli-sensitive cross-links, nanoparticles, growth factor conjugates, antibacterial agents), whereas traditional systems are simpler networks mainly focused on water/solvent uptake and mechanical stability. As noted in recent reviews, the trend is moving toward multifunctional hydrogels combining antibacterial + anti-inflammatory + pro-angiogenic functionalities in one platform.^19^Fig. 1 illustrates a conceptual design of a multifunctional hydrogel for chronic/infected wound healing.

**Fig. 1:**
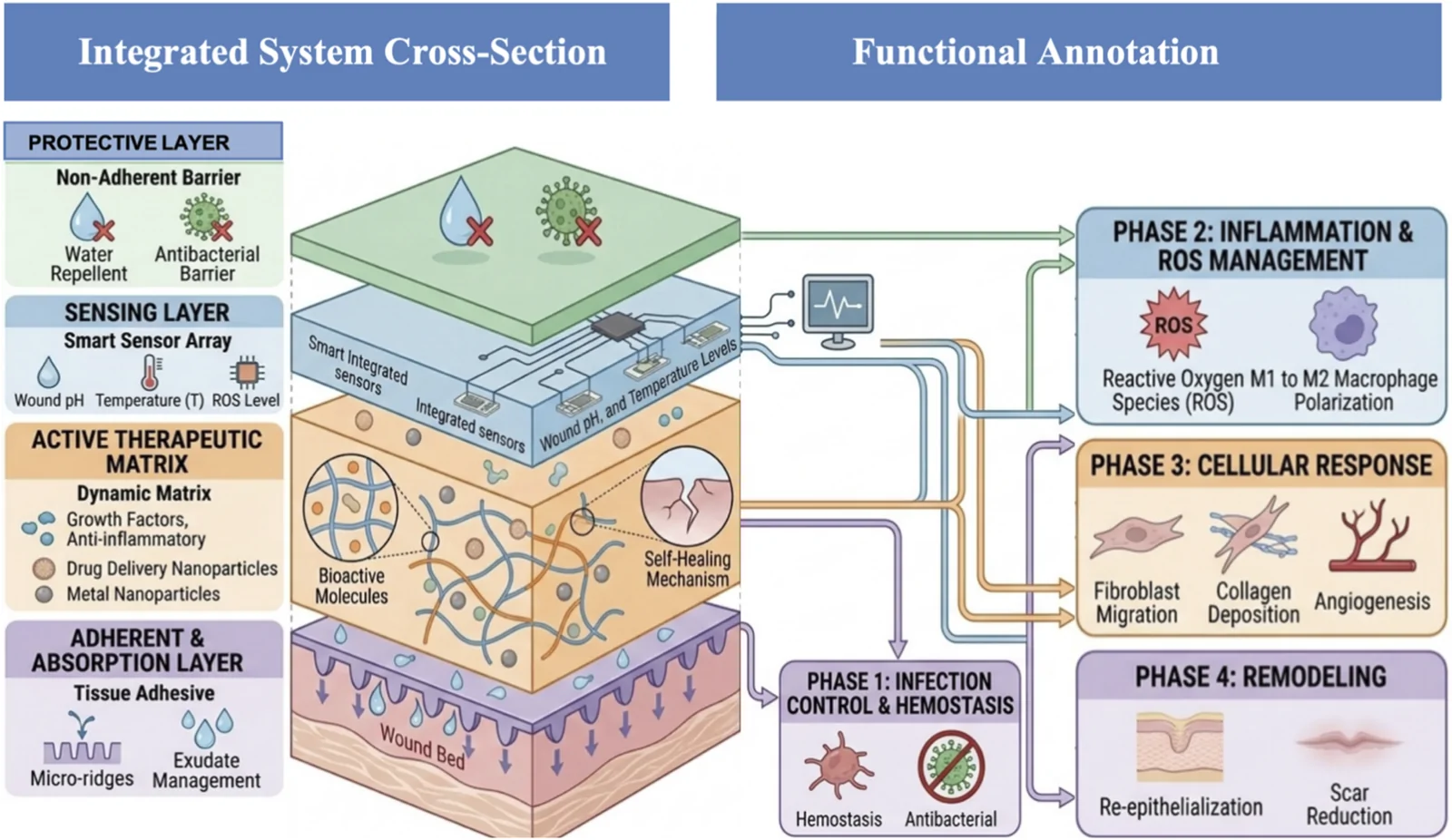
Conceptual design of a multifunctional hydrogel for chronic/infected wound healing, showing the polymer network, embedded antibacterial agents, stimuli-responsive crosslinks, and bioactive cargo release.

### 1.3. Scope, novelty and added value of this review

Recent reviews have extensively described multifunctional, stimuli-responsive and biopolymer-based hydrogels for wound healing. However, the rapid expansion of this field has also produced a fragmented literature in which polymer chemistry, antimicrobial activity, immune regulation, regenerative signaling, advanced fabrication and clinical translation are frequently discussed as separate topics.^20,21^ The present review confronts this fragmentation by formulating a unified paradigm that relates the pathological wound microenvironment with the design of hydrogel networks, therapeutic functionality, and translational effectiveness. Consequently, the key advancement of this review is not the pinpointing of particular hydrogel formulations, but rather the development of a microenvironment material, function, and translation framework. Chronic wounds are characterized by interconnected abnormalities, including persistent microbial burden and biofilm formation, excessive reactive oxygen species (ROS), prolonged inflammatory signaling, dysregulated matrix metalloproteinase activity, impaired angiogenesis and defective extracellular-matrix remodeling. Consequently, a hydrogel designed to address only one of these abnormalities may provide incomplete therapeutic benefit. Smart polymeric systems can instead be designed to couple antimicrobial activity with oxidative-stress regulation, immune modulation, angiogenesis and controlled matrix remodeling. A second distinguishing feature is the emphasis on temporal and spatial coordination of multifunctionality.^22,23^ Antimicrobial activity is particularly important during periods of high bacterial burden, whereas immunomodulation and regenerative stimulation become increasingly important during transition from inflammation to proliferation and remodeling. Stimulus-responsive networks therefore provide an opportunity to synchronize therapeutic release with wound-state transitions rather than delivering all active components simultaneously.^24^ Unlike previous reviews that predominantly classify smart hydrogels according to polymer composition, stimulus responsiveness, or individual therapeutic functions,^25^ this review develops a micro-environment material function translation framework that integrates dynamic network chemistry, antimicrobial immunomodulatory regenerative synergy, AI-assisted design, advanced manufacturing, preclinical model selection and clinical translation. Finally, this review extends the discussion beyond laboratory efficacy by considering dynamic network chemistry, advanced manufacturing, artificial intelligence, wound monitoring, animal-model limitations, regulatory classification, scalability and clinical readiness. This translational perspective is essential because impressive wound-closure percentages in small-animal models do not necessarily predict clinical effectiveness.

### 1.4. Comparison with recent reviews and unique added value of the present review

Recent reviews published during 2024–2026 have provided valuable summaries of multifunctional hydrogels, diabetic-wound hydrogels, smart-response hydrogels and hydrogel-based wound dressings. These studies establish the importance of stimuli responsiveness, antimicrobial activity, immunomodulation, angiogenesis and regenerative medicine.^26–28^ However, differences remain in the depth with which network chemistry, multifunctional synergy, artificial intelligence, manufacturing and clinical translation are integrated. The present review is positioned at the intersection of these themes. Rather than organizing the literature exclusively according to polymer type or stimulus, we organize the discussion according to the biological problems that must be solved in chronic wounds and the material strategies capable of addressing them. This approach enables comparison of how antimicrobial, antioxidant, immunomodulatory and regenerative functions influence one another. Another study compared the stimuli-responsive hydrogel chemistries to three chronic wound microenvironments and concluded with a summary of the potential for using smart hydrogels alongside artificial intelligence but did not explore individual AI techniques.^29^ Zhang *et al.* listed the individual properties of the hydrogels: adhesion, anti-bacterial property, hemostasis, promotion for angiogenesis, anti-oxidation, anti-inflammatory effect, self-healing and conductivity as the independent design properties.^30^ Srivastava *et al.* performed a review of the commercially available products available in the market in Europe, North America, and Asia based on natural compounds, stem cell, and microRNAs, which provided market and regulatory perspective but lacked mechanistic design.^31^ The present review differs from each of these in three specific ways: (i) it explicitly addresses the mechanistic crosstalk between the antimicrobial, immunomodulatory and regenerative functions of hydrogel systems, rather than assuming they are parallel design tracks; (ii) it reviews and summarizes published examples of named hydrogel systems, and translates these into a single table that makes the technology readiness of each system more concrete and clearly visible against the benchmark of the other two functions; and (iii) it goes beyond a brief closing outlook to critically analyze the technology readiness of AI- and biosensor-integrated hydrogel systems against concrete examples of published hydrogels.

## 2. Multifunctional hydrogels: material strategies & mechanisms

### 2.1. Material design strategies

The selection of natural and synthetic polymers is the first step in the design of multifunctional hydrogels. While synthetic polymers like PEG and PNIPAM give more control over molecular architecture, mechanical characteristics, and stimulus reactivity, natural polymers like chitosan, alginate, gelatin, and hyaluronic acid offer biodegradability, hydration, and cell-interactive motifs. These polymeric components can be assembled using physical, covalent, or dynamic cross-linking techniques to control network stability, degradation, swelling, and therapeutic release.^32^ Physical cross-linking relies on reversible interactions such ionic attraction, hydrogen bonding, hydrophobic association, or host–guest recognition, whereas chemical cross-linking produces more robust covalent networks. Dynamic covalent chemistries, like Schiff-base/imine, boronate ester, and disulfide linkages, provide an intermediate approach that allows self-healing and stress alleviation while maintaining improved structural cohesion, in contrast to many purely physical networks. As a result, the choice of cross-linking method influences not just drug release and mechanical strength but also the dynamic mechanical environment that cells experience.

Multifunctionality can also be achieved by adding antimicrobial peptides, growth factors, stem cells, extracellular vesicles, nanoparticles such as silver, ZnO, or MnO_2_, or other bioactive components. In order to enhance mechanical performance while maintaining cellular accessibility, network design can be further developed using interpenetrating networks (IPNs), semi-IPNs, double networks, nanofiber reinforcement, and spatially patterned structures. Hydrogel-tissue interactions can be further controlled by stimuli-responsive functional groups, such as catechol for tissue adhesion and Schiff-base linkages for pH-responsive network remodeling.^33^ Furthermore, these design criteria ought to be seen as biological regulators instead of merely material-engineering factors. The composition of polymers, crosslink density, mesh dimensions, viscoelastic properties, ligand display, surface topography, and degradation rates jointly characterize the three-dimensional microenvironment perceived by both resident and invading cells. Consequently, material design must strive to attain sufficient mechanical integrity and therapeutic delivery while also creating a temporally and geographically suitable extracellular-matrix-like milieu that facilitates cell migration, immunological modulation, angiogenesis, and tissue regeneration. Table 2 summarized representative multifunctional hydrogel design features and their targeted wound-healing functions.

**Table 2:** Representative multifunctional hydrogel design features and wound-healing functions

Design feature	Functional component	Targeted wound-healing role	Ref.
Silver nanoparticles/cationic polymer	Antimicrobial agent	Preventing biofilm/infection	34
ROS-scavenging moiety (MnO_2_)	Anti-oxidative stress	Reducing ROS in chronic wound	35
Growth factor (EGF, VEGF) loaded	Pro-angiogenic	Stimulating angiogenesis, granulation	36
Stimuli-responsive crosslinks (*e.g.*, pH/ROS)	Smart release network	Releasing on demand release in responsive wound micro-environment	37
Adhesive moieties (*e.g.*, dopamine)	Tissue adhesion	Conform to irregular wounds, reduce detachment	38

Multifunctional hydrogels act across multiple wound-healing phases during hemostasis/inflammation, hydrogels with hemostatic adhesives or bioactive moieties may reduce bleeding, absorb exudate and begin to regulate inflammatory responses. In the proliferation phase, the hydrogel scaffold supports fibroblast migration, ECM deposition, angiogenesis and re-epithelialization. Embedded cues (growth factors, stem cells) help accelerate granulation tissue formation. In the remodeling phase, hydrogels that degrade at controlled rates allow replacement by native tissue while minimizing scar formation.^39^

## 3. Classification of hydrogels on basis of nature

### 3.1. Natural and synthetic hydrogels

Polysaccharides like alginate, chitosan, agarose, HA, cellulose, *etc.*, or proteins obtained from biological sources (such collagen, gelatin, elastin, fibrin, *etc.*) are used to make natural hydrogels. Natural hydrogels are low-toxicity, biodegradable substances with good biocompatibility. It is preferable to use hydrogels made of natural polymers rather than synthetic ones. They are mostly utilized in a variety of food applications, such as packaging, medicine delivery, encapsulation, and, more recently, the creation of structured meals.^40^ Hydrogels made by chemically polymerizing synthetic monomers are known as synthetic hydrogels. Poly(ethylene glycol) diacrylate, poly(acrylic amide), and poly(vinyl alcohol) are examples of synthetic hydrogels. Many synthetic hydrogel systems have historically included linearly elastic networks of high molecular-weight polymers, which provide engineers considerable control over the cell microenvironment but do not physiologically absorb or distribute stresses produced by cells. To change synthetic polymers from inert physical supports into bioactive matrices that may interact with encapsulated cells, they usually need to undergo chemical changes, such as conjugation of integrin-binding motifs or the addition of hydrolytically labile or enzyme-cleavable connections.^41^

### 3.2. Hybrid polymer networks

Hybrid and multicomponent hydrogels are increasingly important because no single polymeric network can simultaneously optimize mechanical strength, hydration, degradation, bioactivity, stimulus responsiveness and therapeutic delivery. Combining materials with complementary properties allows individual weaknesses to be compensated through network-level design. However, increasing compositional complexity also introduces additional interfaces, potential incompatibilities, manufacturing challenges and regulatory concerns.^42^ Hybrid hydrogels commonly combine natural polymers such as gelatin, collagen, chitosan, alginate or hyaluronic acid with synthetic polymers such as PEG, PVA, PNIPAM or polyacrylamide derivatives. Natural polymers offer cell-interactive features and are biodegradable, while synthetic polymers enable enhanced regulation of molecular weight, crosslink density, mechanical characteristics, and degradation rates.^43^ Importantly, the polymer network should not be seen solely as a structural scaffold or a storage medium for medicinal compounds. The network constitutes a three-dimensional biophysical environment that can dynamically influence cellular functions.

Numerous parameters, like as crosslink density, network connectivity, mesh size, ligand presentation, fibrillar form, and viscoelastic stress relaxation, affect how cells perceive and react to the matrix. Elevated crosslink density typically enhances elastic modulus while reducing mesh size and swelling, whereas loosely crosslinked networks allow more molecular mobility but may not have enough mechanical stability.^44^ These modifications also affect the mechanical signals carried by cell–matrix adhesions and the extent to which cells can bend and reorganize their surrounding matrix. Therefore, molecular transport, cellular spreading, migration, cytoskeletal architecture, proliferation, and differentiation can all be regulated by the mechanical properties of a hydrogel. This idea suggests that hydrogels should not be thought of as inert carriers but rather as active biophysical switches. Cell–matrix mechanotransduction converts physical inputs such matrix stiffness, viscoelastic properties, surface texture, spatial restriction, and the relaxation of mechanical stress into intracellular signals that can affect gene expression and cellular state. As a result, a wound hydrogel can be engineered to change its mechanical characteristics in line with the biological phases of healing: a cohesive framework at first can ensure protection and structural integrity, while later relaxation, degradation, or reversible bond exchange can improve cellular accessibility and promote matrix deposition and remodeling.

This idea has a special biological significance at the wound boundary, where keratinocytes, fibroblasts, endothelial cells, macrophages, and other stromal cells interact with a spatially heterogeneous and continually changing extracellular matrix. While matrix stiffness and viscoelasticity can have an impact on macrophage phenotype, fibroblast activity, epithelial migration, and vascular organization, sticky ligands and fibrillar structures provide additional biochemical and structural data. As a result, optimizing a hydrogel only on its bulk elastic modulus may be insufficient. For a more physiologically accurate design, the interrelated interactions between stiffness, stress relaxation, ligand density, matrix degradability, pore architecture, and cellular remodeling capacity should be studied. Such structures could be especially advantageous for injectable and printable wound hydrogels, where shear-thinning, fast recovery, and post-deposition remodeling must coexist with cellular compatibility. Structured scaffold techniques have shown that oriented channels, patterned patterns, and controlled microenvironments can influence cellular organization and tissue regeneration.^45^ As a result, the next generation of hybrid hydrogels should be designed to be adaptable extracellular matrix-like environments that correspond to the temporal stages of wound healing, rather than multipurpose drug-delivery matrices. This allows chemical, mechanical, and structural cues to be coordinated. Fig. 2 illustrates the dual use of hydrogels in tissue engineering scaffolds and medication delivery systems.

**Fig. 2:**
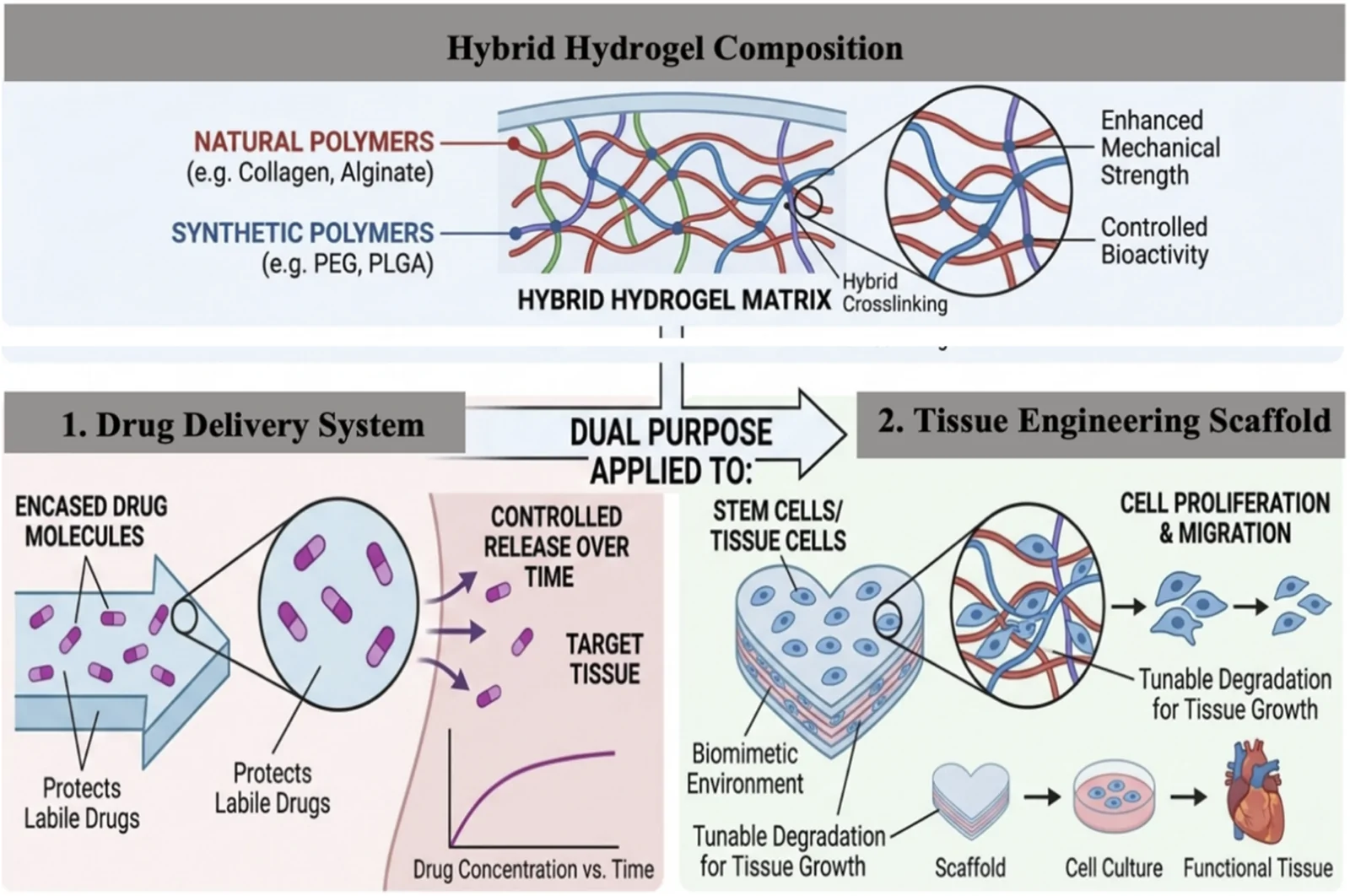
Drug delivery systems and tissue engineering scaffolds are two uses for hybrid hydrogels, which are made of a blend of synthetic and natural polymers.

### 3.3. Crosslinked nanogels, and swelling, degradation and release kinetics of hydrogels

To create nanoscale reservoirs for medications, proteins, or bioactive compounds, nanogels can be integrated into macroscopic hydrogel networks. They can enhance loading and release control due to their large surface area and adjustable surface chemistry. On the other hand, swelling, diffusional transport, and mechanical relaxation can all be significantly altered by the interaction between nanogels and the surrounding network. Fe_3_O_4_ nanoparticle-containing gelatin/vinylic semi-IPN magnetic hydrogel beads with adjustable morphology, rheology, swelling, and cell attachment were created by the microfluidic investigation.^46^ Hydrogels with nanoparticles offer an additional path to multifunctionality. While magnetic nanoparticles can offer mechanical reinforcement and distant responsiveness, metal and metal-oxide nanoparticles can give antibacterial activity. However, as increasing nanoparticle loading may result in increased cytotoxicity, aggregation, or long-term retention rather than a proportionate therapeutic effect, nanoparticle concentration needs to be optimized. According to recent research, amine-functionalized superparamagnetic maghemite nanoparticles can improve thixotropic behavior, alter hydrogel rheology, and facilitate drug release that is dependent on magnetic fields. These systems demonstrate how nanoparticles can serve not only as passive antibacterial additions but also as therapeutic-control components and structural modifiers. Another study demonstrated the use of UV-triggered 3D micro extrusion printing to create a borophene/ZnO nanocomposite integrated into a semi-interpenetrating polymer network.^47^

Polymers, nanoparticles, peptides, growth factors, cells, exosomes, and small-molecule medications can all be found in multicomponent hydrogels. The ability to address several pathological hurdles at once is their main advantage. But increasing the number of components shouldn't be the only goal of the design. Every element must have a distinct therapeutic function and a quantifiable impact on the ultimate biological result. An immunomodulatory signal to encourage inflammatory resolution, an antimicrobial component to lessen bacterial burden, an antioxidant or ROS-responsive component to lessen oxidative stress, and an angiogenic/regenerative cue to support tissue reconstruction can all be combined in a logical architecture for chronic wounds. The components may also be spatially separated to prevent undesirable interactions. Simultaneous release of antimicrobial, anti-inflammatory and regenerative factors may not reproduce the physiological sequence of healing. The multilayer or stimulus-responsive networks can offer early antibacterial activity followed by delayed immunomodulatory and regenerative activation,^48,49^ they might be preferred. The dynamics of swelling, breakdown, and release are important factors that affect hydrogel performance. The effective transport routes for soluble molecules can be expanded and polymer-chain mobility is increased by water uptake. On the other hand, rapid loss of therapeutic payloads and a reduction in mechanical integrity might result from severe edema. On the other hand, a high crosslink density can limit cell migration and decrease drug diffusion while simultaneously reducing swelling and improving mechanical stability. Fickian diffusion, swelling-controlled transport, degradation-controlled release, and stimulus-triggered network cleavage are just a few of the interacting mechanisms that might result in drug release. These methods may work concurrently for systems with many functions. Therefore, a predictable release profile should be the design goal instead than just a high cumulative release percentage.^50^

### 3.4. Based on stimuli responsiveness

#### 3.4.1. pH-Responsive, thermosensitive, enzymes and redox responsive, magnetic field responsive hydrogels

The pH-dependent responsiveness derives from the ionization equilibrium of functional groups, which modifies the osmotic pressure and electrostatic repulsion inside the polymer network and ultimately regulates the hydrogel's macroscopic behaviour. pH-Responsive hydrogels vary in size and swelling according to the pH of their environment.^51^ They are mainly classified as polybasic (cationic) and polyacidic (anionic) hydrogels. Polybasic hydrogels, which inflate at lower pH as ionization rises, aid in drug release as shown in Fig. 3.

**Fig. 3:**
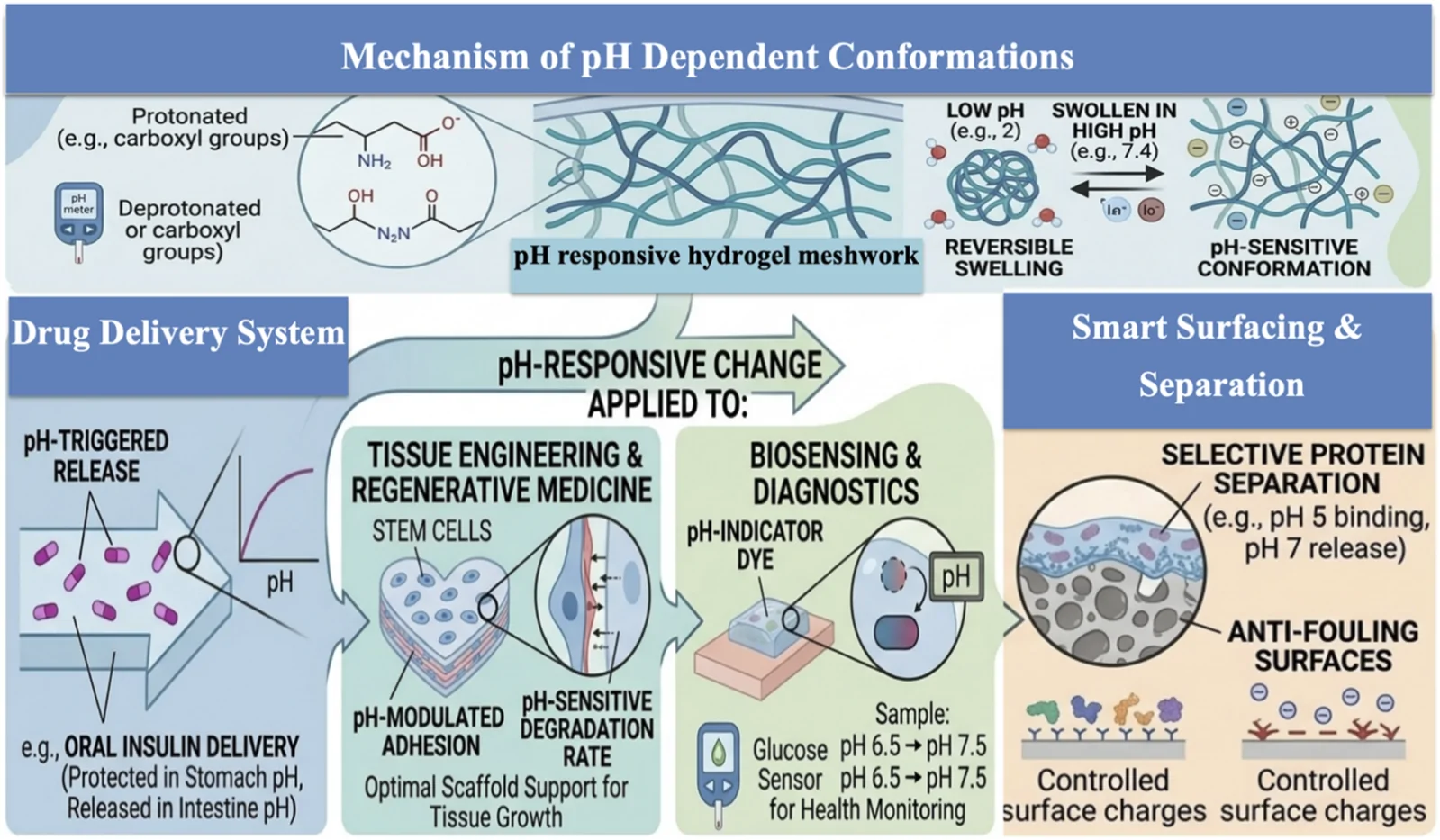
Applications of pH responsive hydrogels in drug delivery system.

Temperature-sensitive hydrogel designs contain both hydrophilic and hydrophobic elements. Hydrogels exhibit separation between solution and solid at either the upper critical solution temperature (UCST) or the lower critical solution temperature (LCST). The polymer is soluble under the LCST, but when the temperature rises, it turns hydrophobic and insoluble, forming a gel.^52^ Supramolecular motifs are inherently temperature-sensitive since high temperatures usually prevent them from developing and/or accelerate their dissociation. Therefore, by molecularly constructing supramolecular recognition and selecting a particular motif or polymeric/macromeric backbone, it is possible to create a variety of supramolecular hydrogel materials to enable a range of prospective medicinal applications.^53,54^ Numerous pharmaceutical applications, including anti-tumor drug administration, transdermal drug delivery, nasal drug delivery and buccal drug delivery as shown in Fig. 4.

**Fig. 4:**
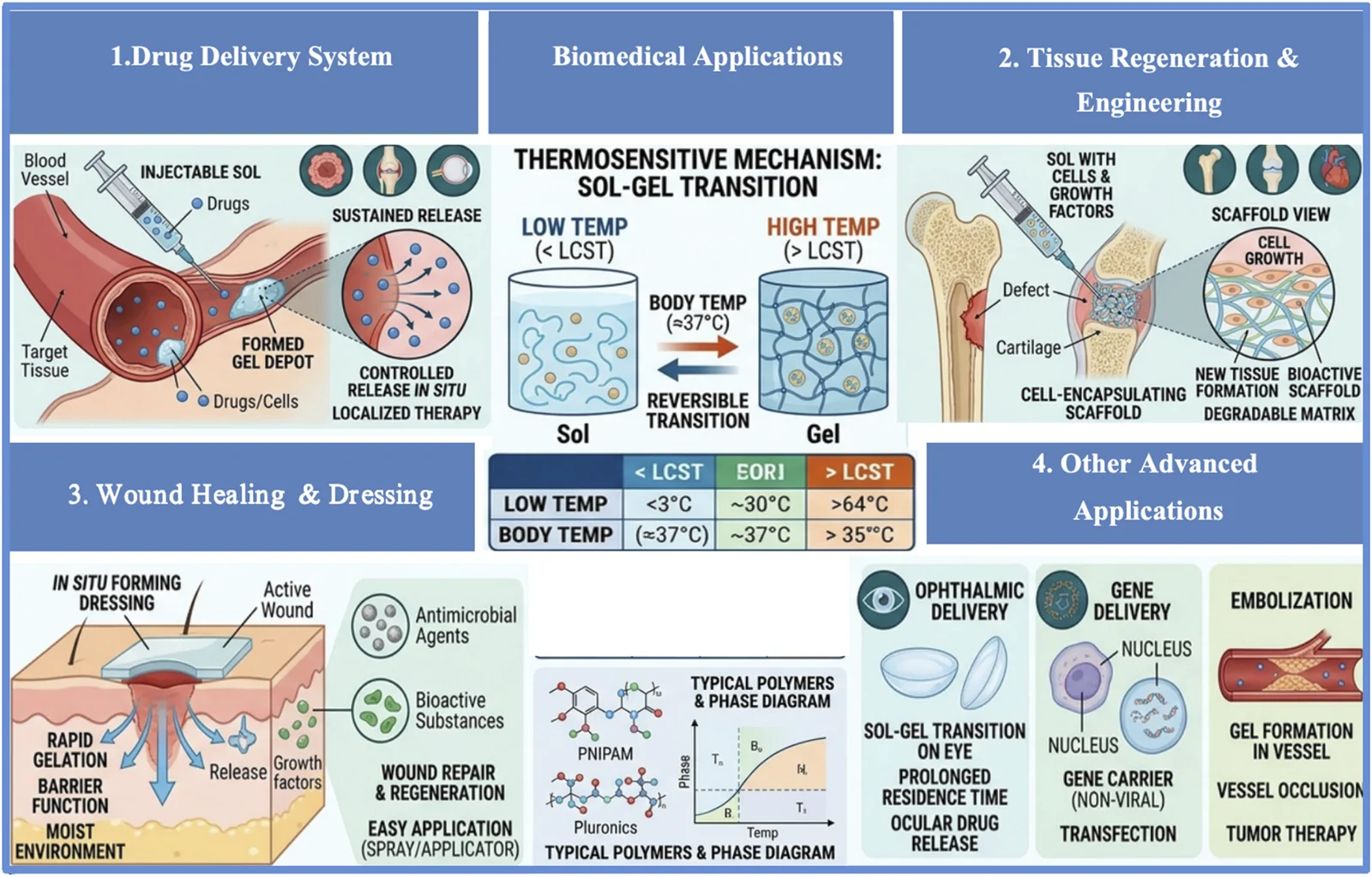
Applications of thermosensitive hydrogels.

The main method for the enzymatic synthesis of healable polymeric hydrogels is self-assembly and polymerization. Additionally, reversible covalent bonds (such acyl hydrazone, disulphide, borate ester, and imide bonds) that can be broken down in response to stimuli and subsequently reformed to give hydrogels the required properties can be created using enzymes. Additionally, enzymatic breakdown is necessary for the injected or implanted hydrogels to be cleared autonomously and non-invasively.^55^ The functions and mechanism of hydrogels are shown in Table 3.

**Table 3:** Overview of the enzymatically fabricated healable polymeric hydrogels

Functions	Enzymes	Driving forces	Ref.
Enzymatic polymerization	Acid phosphatase (AP), glucose oxidase (GOX), horseradish peroxidase (HRP)	Hydrogen bonding, π–π stacking	56
Enzyme-mediated reversible covalent bonds	Monoamine oxidase B (MAO B), catalase (CAT), plasma amine oxidase (PAO), urease	Imide bond, hydrogen bonding, acyl hydrazone bonds	57
Enzyme assisted carbon dioxide fixation	Glucosidase, GOX	Carbon fixation, glucose export, polymerization	58

Redox-responsive hydrogels were produced by combining linear PEG polymers containing reactive PDS groups at the ends of their chains with tetra-arm PEG-thiol macromonomers. These hydrogels can swell or de-swell dramatically in response to even small changes in external variables including temperature, pH, ionic strengths, solvent composition, and light intensity. Due to their volume phase transition temperature (VPTT) of approximately 32 °C, which is near the physiological temperature, hydrogels based on poly(*N*-isopropylacrylamide) (PNIPA) have been the focus of the most research in recent decades.^59^ The hydrogel's drug release behaviour demonstrated dual reactivity to both pH and static magnetic field, as well as dual parameter dependency (IONPs and cross-linker). This hybrid hydrogel may be a perfect substitute for the remotely controlled drug delivery vehicle due to its delayed network rupturing, dual-responsive drug delivery nature, and noncytotoxic behaviour against human live cells.^60^ They can greatly increase local temperatures by thermal-ablation processes when subjected to a magnetic field, which improves therapeutic efficacy. Furthermore, these devices are usually connected to thermosensitive hydrogels, which release medications in response to temperature increases, increasing the combined effectiveness of thermal and chemotherapeutic cytotoxicity.^61^

## 4. Based on physical form and application

### 4.1. Injectable, sprayable, self-healing, adhesive and shape forming hydrogels

The fundamental idea underlying injectable hydrogels is that some biomaterials can be injected as liquids into the human body to create an *in situ* solid hydrogel. To overcome the short half-life of HA, which is only a few hours in the body due to its rapid enzymatic degradation by hyaluronidase, chemical crosslinking compounds like divinyl sulfone (DVS) and 1,4-butanediol diglyceryl ether (BDDE) are utilized to make HA-based injectable hydrogels.^62^ Biomass materials are frequently used to create sprayable hydrogels due to their easy accessibility, biocompatibility, biodegradability, and other physicochemical characteristics.^63^ Because sprayable hydrogels gel in place as they come into contact with tissue, they can be sprayed smoothly to even complex surfaces. In tissue engineering, drug administration, and wound care, where localized and sustained drug release is essential, this feature is especially beneficial.^64^

Self-healing hydrogel has shown promising applications in tissue engineering, medicine delivery, 3D/4D bioprinting, and 3D cell culture. For the delivery of cells during heart regeneration, conductivity-enabled injectable self-healing hydrogels are perfect. The processes of self-healing hydrogels are based on the reversibility of crosslinking, which includes hydrogen bonding, supramolecular host–guest interactions, ionic, hydrophobic, and dynamic covalent connections.^65^ Furthermore, since these properties are mostly established in non-physiological settings, it would be challenging to verify that hydrogels' well-established self-healing properties are maintained in physiological settings, such as with electrolytes, under mechanical stress, and in the presence of material–cell interaction. Reversible crosslinks, as opposed to permanent ones, are readily broken to encourage biodegradation and to limit biodegradation by repairing damage at both the macro and micro scales.^66^ The main factors influencing hydrogel adhesion include bond chemistries, connection topologies, and energy dissipation mechanisms. Strategies like surface modification, surface initiation, bulk modification, bridging polymers, topological adhesion, and the use of nanocomposites have all been developed to create strong hydrogel adherence. In nanocomposite hydrogels, nanoparticles and polymer chains generate strong linkages that improve adhesion by lowering adhesion energy and providing energy dissipation.^67^ Sticky hydrogels with intrinsic tissue adhesiveness have enormous potential for treating osteoarthritis. They can stay steady at the application location.^68^

## 5. Multifunctional properties of smart hydrogels

### 5.1. Antimicrobial functions

Antimicrobial-containing hydrogels have the amazing ability to stop or treat microbial infections.^69^ By modifying the capsules' chemical makeup and structure, the antibacterial qualities of these structures can be modified. For instance, polymer nanoparticles coated with cationic amino cellulose and anionic glycosaminoglycan-hyaluronic acid were created. In a different study, scientists created alginate and polycyclodextrin microcapsules with quaternary ammonium moieties as antibacterial agents. The produced microparticles showed an inhibitory effect on the growth of *S. aureus* and *E. coli*.^70,71^ Hydrogels for functionalizing drug carriers with antimicrobial coatings in a variety of therapeutic applications are elaborated in Fig. 5.

**Fig. 5:**
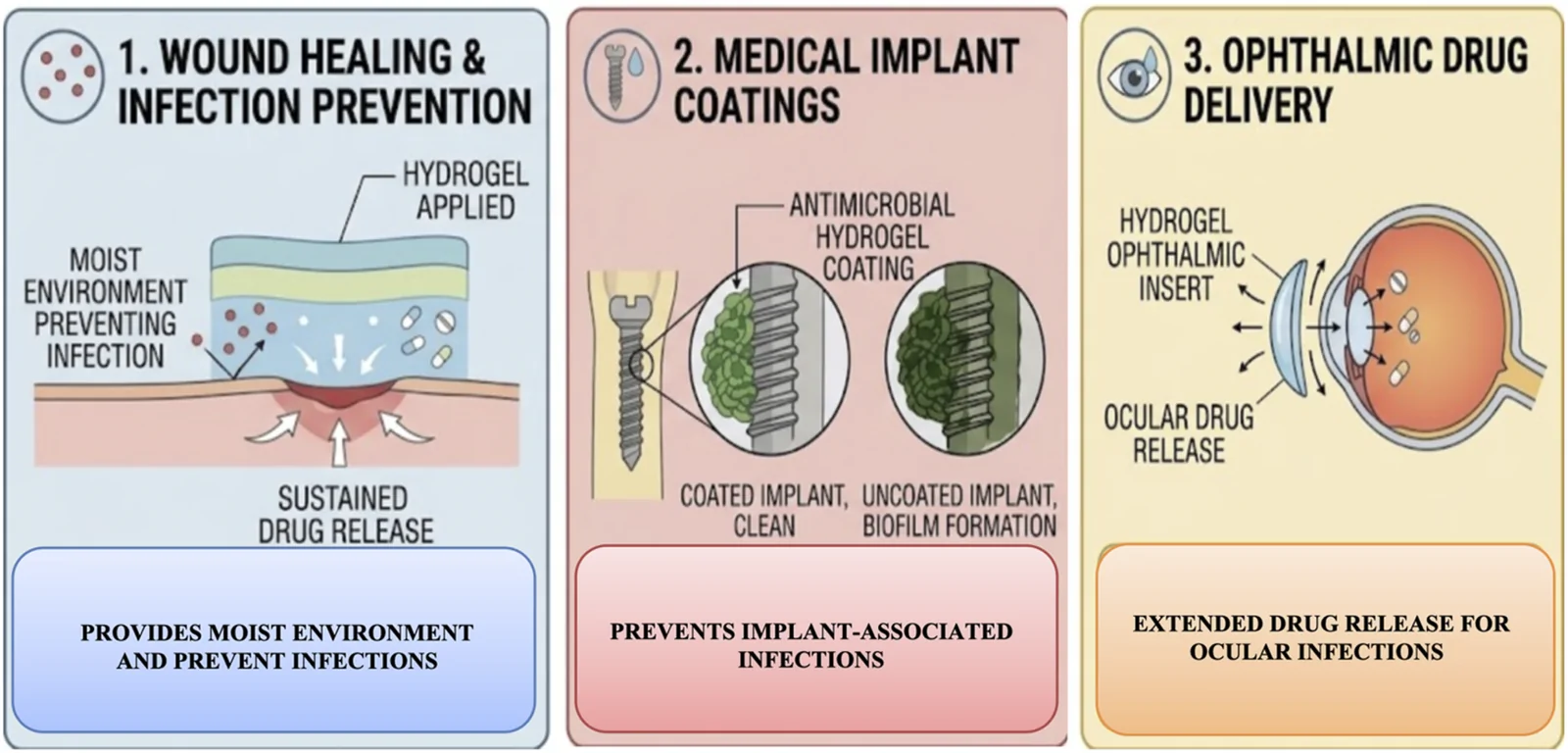
Use of antimicrobial hydrogels in chronic wound dressings, medical implant coatings, prevents biofilm infections, ophthalmic drug delivery.

Antimicrobial hydrogels are incredibly attractive materials that are frequently created by (i) covalently ligating active chemicals through self-assembly mechanisms or (ii) loading them with pharmaceutical receivers (drugs).^72^ It has been demonstrated that chitosan inhibits the growth of filamentous fungus, bacteria, and yeast strains. Against both Gram-positive and Gram-negative bacteria, chitosan exhibits a high death rate and a wide range of activity. The antibacterial qualities of six chitosan oligomers with wildly disparate molecular masses were examined by Hamedi *et al.* Gram-positive and Gram-negative microbes were employed to test this theory. They found that chitosan significantly reduced the development of most of the bacteria they examined, however the effects differed according to strain and molecular size. Gram-positive bacteria were more susceptible to the bactericidal effects of chitosan than Gram-negative bacteria.^73^

### 5.2. Immunomodulatory functions

Materials based on hydrogel have been employed as responsive wound dressings because they can interact with skin tissues to promote healing and adjust to the conditions of the wound microenvironment. For instance, hydrogels generated from GAG, like HA, are known for their interaction qualities in dressings that promote wound healing.^74^ Mechanism of actions of immunomodulatory hydrogels are illustrated in Fig. 6.

**Fig. 6:**
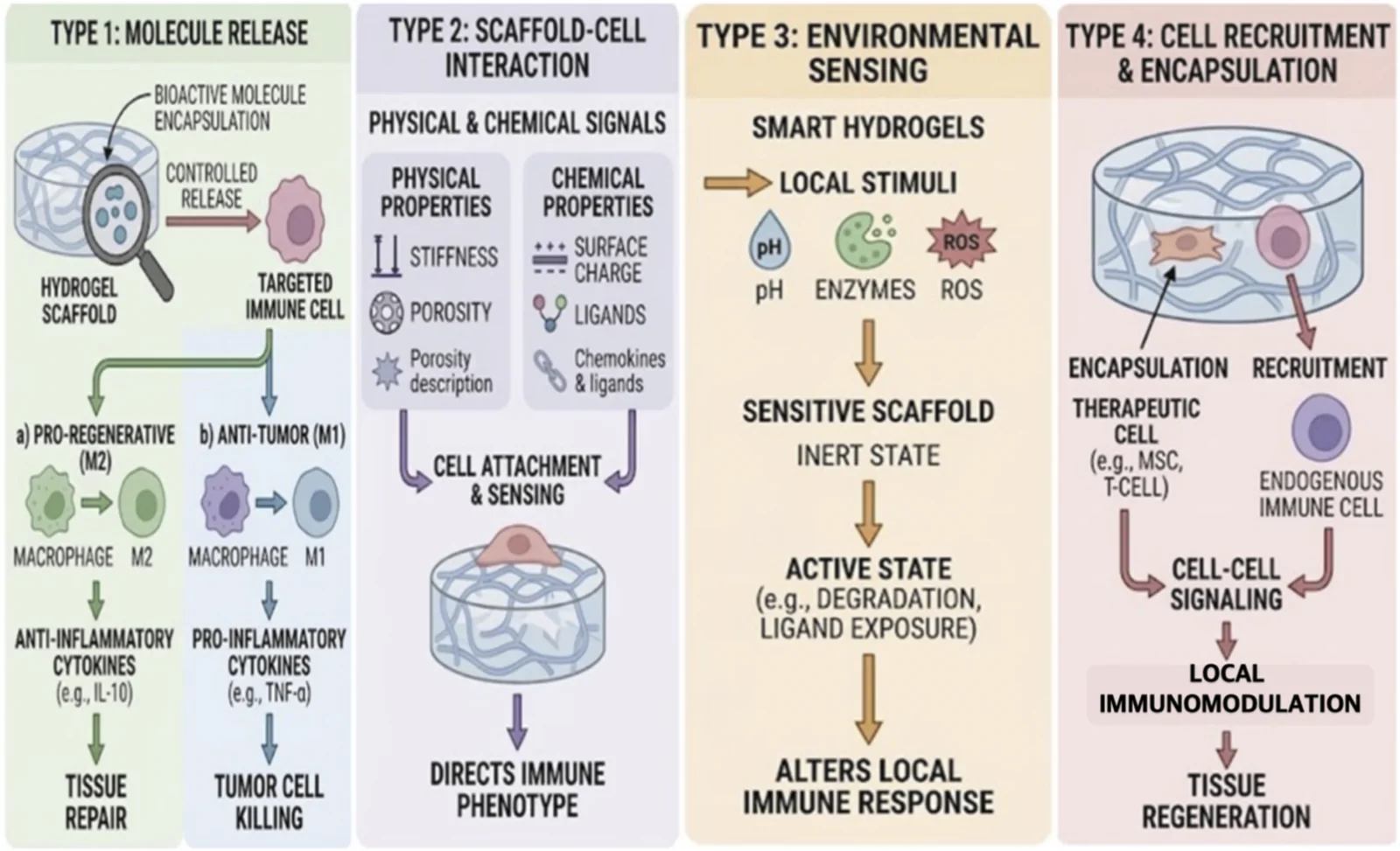
Mechanism of immunomodulatory hydrogels including release, interactions, scaffolding, and therapeutic aspects.

Hydrogel, hydrogel–cell, hydrogel–molecule, hydrogel–exosome, and combination systems are among the hydrogel systems being used for tissue regeneration. It was discovered that when macrophages were cultivated on medium-stiff hydrogels as opposed to those on low-stiff hydrogels, they expressed more CD206 and secreted more IL-4 and TGF-β. Since mild stiffness might also cause M2 macrophage polarization *via* the ROS-initiated NF-κB signaling pathway, the underlying mechanism was clarified.^75^ By releasing agents that inhibit monocyte migration or macrophage maturation, impeding macrophage infiltration, scavenging toxic products from activated macrophages, such as reactive oxygen species, promoting an M2-like macrophage phenotype, preventing activated T cell migration from lymph nodes, and inducing immune cell apoptosis, hydrogels can control inflammation.^76^ Encasing drug-loaded mesoporous silica nanoparticles (MSNs) in a ROS-responsive hydrogel is an efficient method for intelligent nanoparticle release in the ROS-enriched inflammatory microenvironment caused by post-implantation immunological rejection. On-demand drug release is made possible by this nano reservoir system's breakdown in reaction to ROS.^77^

### 5.3. Biosensing and diagnostic functions

The following are some of the benefits that hydrogel-based systems have demonstrated: (i) increasing the loading capacity of the probe and analyte; (ii) preventing nonspecific biomolecule adsorption; (iii) reducing background signal; (iv) permitting modulation of the biosensing molecules; (v) enabling the combination of sensing with transducing moieties; (vi) offering a highly aqueous environment that is biocompatible; and (vii) acting as a matrix for sample collection and storage.^78^ A biochemical mechanism is used by a bioreceptor, which can be a biological system like a cell or an organ, or a molecular species like an enzyme, antibody, gene, or nucleic acid, to interact with the analyte as shown in Fig. 7. In contrast, a transducer generates a detectable signal in proportion to the interaction between the bioreceptor and the analyte. Base materials are linked with bioreceptors using various immobilization procedures. Metals, polymers, glass, and composites can all be used as base materials, and are often selected because of their remarkable physicochemical and optical properties.^79^

**Fig. 7:**
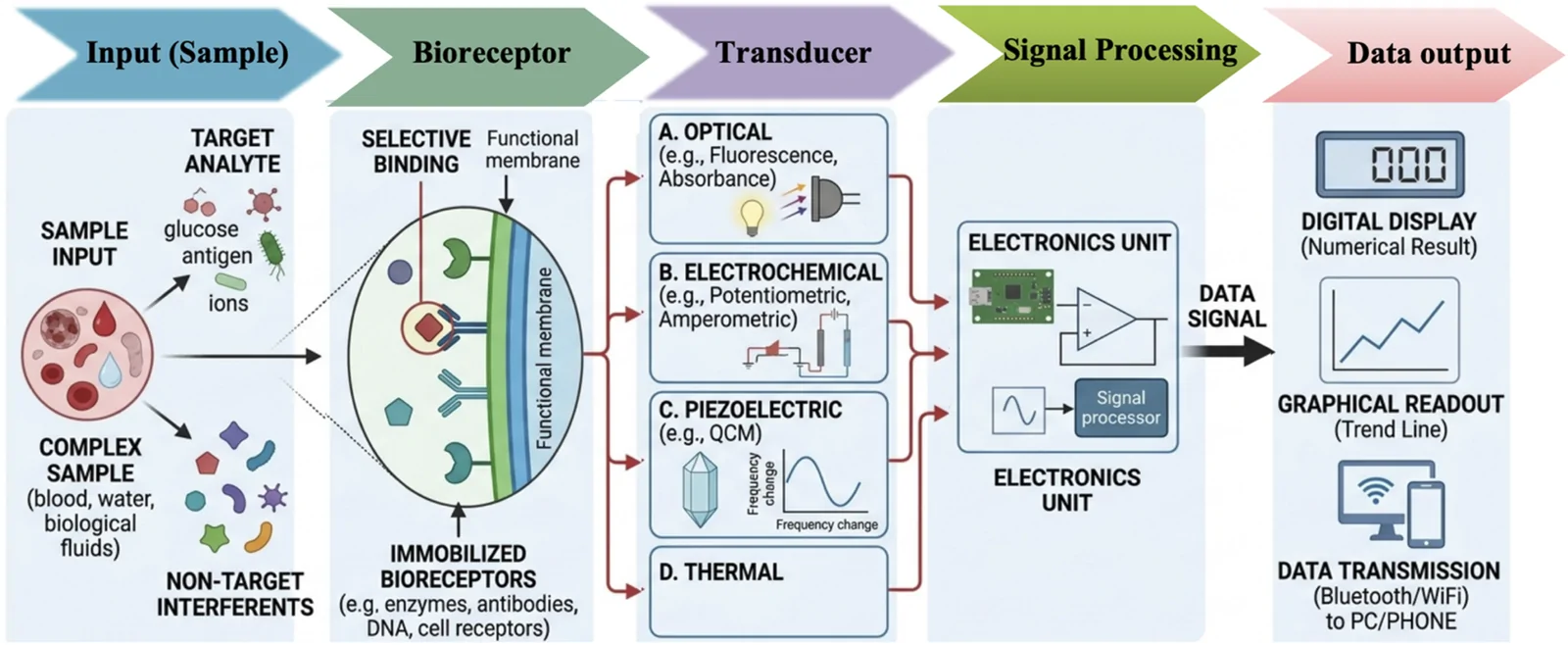
Structure of biosensor system for diagnostic purposes used in the hydrogels.

A typical biosensor system consists of materials, structural components, and sensing techniques. A hydrogel-based biosensor can be created using hydrogel as the basis material since it is a biocompatible polymer with a high capacity for water absorption, and it can be paired with the aforementioned measurement techniques (conductometric, potentiometric, amperometry, impedimetric, surface charge, piezoelectric, magnetoelastic, surface acoustic wave, fiber optic, absorbance, and luminescence).

## 6. Fabrication techniques and material design

### 6.1. Crosslinking strategies: physical *vs.* chemical, hybrid, and dynamic bonds

Cross-linking is a critical factor in the formation of hydrogels; however, its biological significance extends beyond the regulation of mechanical strength, enlargement, degradation, and therapeutic release. The dynamic mechanical environment that cells are exposed to is determined by the type, density, reversibility, and spatial organization of crosslinks. Consequently, cross-linking chemistry should be regarded as a method for stabilizing a hydrogel and a means of creating a microenvironment that is similar to an extracellular matrix-like cell-instructive environment. In this context, the hydrogel network can serve as a dynamic biophysical switch that transforms variations in biochemical and mechanical conditions into cellular responses. Physical cross-linking is accomplished through reversible non-covalent interactions like hydrogen bonding, ionic attraction, hydrophobic association, and host–guest interactions. Because these interactions can occur under relatively benign conditions, they are advantageous for injectable hydrogels and systems including cells, proteins, or other sensitive biological components.^80,81^ Because of the quick dissociation and reformation of individual non-covalent connections, physically crosslinked networks can exhibit shear-thinning, self-healing, and stress-relaxation behavior in addition to efficiently dissipating mechanical energy. However, when exposed to physiological ionic strength, dilution, or prolonged mechanical loading, their reversibility may also result in a reduction in long-term mechanical stability. As a result, physical cross-linking creates an extremely dynamic network; nonetheless, reinforcing can be required to preserve structural integrity over time.

Photo-polymerization, enzymatic processes, click chemistry, and other covalent coupling reactions *via* chemical cross-linking are some of the mechanisms that produce covalent networks. Permanent covalent bonds typically have better structural stability, elasticity, and resistance to deformation than physical crosslinks. Photo-crosslinking is especially useful for *in situ* gelation and spatially controlled manufacturing because methacrylated polymers, such as GelMA and methacrylated hyaluronic acid, can be polymerized either during 3D bioprinting or after implantation at the target region.^82^ However, very persistent and densely crosslinked networks can limit molecular transport and cellular remodeling if the network is overly rigid or resistant to breakdown. Therefore, strong mechanical strength should not be intrinsically linked to biological performance. Reversible physical interactions and dynamic covalent crosslinks differ significantly. Although both are capable of producing adaptive behavior, their molecular mechanisms and relaxation kinetics differ. Due to mechanical deformation or changes in the surrounding environment, physical interactions which are non-covalent can rapidly dissolve. This rapid bond exchange facilitates effective energy dissipation and stress relaxation. Conversely, reversible covalent chemistry distinguishes dynamic covalent bonds, which typically offer better network connections while maintaining the ability to swap or break bonds. Disulfide, boronate ester, Schiff-base/imine, and related reversible chemistries are examples of dynamic covalent systems that are representative.^83,84^ While diol-containing species and pH can influence boronate ester exchange, Schiff-base formation between aldehyde and amine groups can lead to self-healing and pH-sensitive network remodeling. Disulfide connections provide an additional route for environmentally sensitive network remodeling since they are resistant to redox-mediated exchange.^85^

Furthermore, the viscoelastic behavior of wound hydrogels is particularly affected by this differential. A rapidly exchanging physical network may undergo significant stress reduction over relatively short periods of time, but a dynamic covalent network might maintain a higher degree of structural integrity while allowing a progressive redistribution of stress.^86^ Both processes can be combined in hybrid systems to ensure that a stable covalent network maintains macroscopic integrity, even while reversible physical or dynamic covalent interactions release mechanical energy and permit local network rearrangement. By separating toughness from stiffness, these topologies enable the independent tuning of viscous and elastic responses. This is especially crucial because, instead of coming into contact with a hydrogel as a single elastic modulus, cells constantly interact with a matrix whose deformation, relaxation, and remodeling properties influence the mechanical feedback they receive.

#### 6.1.1. Crosslinking as a regulator of cellular mechanotransduction

When the hydrogel manifests as a dynamic extracellular matrix instead of a passive carrier, the biological significance of cross-linking becomes apparent. Cell adhesion and matrix remodeling are influenced by matrix stiffness, viscoelasticity, stress relaxation, pore size, ligand accessibility, and degradability. Integrin-mediated adhesion complexes and cytoskeletal structures enable cells to identify these characteristics and translate external mechanical input into internal signals. Changes in cytoskeletal tension and adhesion signaling can affect cell migration, proliferation, differentiation, inflammatory phenotype, extracellular matrix deposition, and spreading.^87^ Therefore, when maximizing cross-linking density, the necessary bulk modulus should not be the only factor taken into account. A decrease in mesh size and an increase in network stiffness are common outcomes of increasing crosslink density, which may improve structural stability and reduce molecular diffusion. However, excessive cross-linking may limit cell-mediated matrix deformation, cellular infiltration, and remodeling. Although it might not provide sufficient mechanical integrity, a loosely crosslinked network could aid in matrix reconfiguration and cellular migration. In the biologically appropriate design area, stability and cellular accessibility, structural integrity and remodeling, and stiffness and stress relaxation must all be balanced. The significance of crosslinking-dependent mechanics in wound healing is further supported by recent research. Network chemistry can indirectly affect the biological connections required for matrix remodeling and inflammation resolution, as demonstrated by the impact of hydrogel cross-linking on macrophage activity, fibroblast responses, and macrophage–fibroblast communication during tissue regeneration. Similarly, as biomimetic and immunomodulatory hydrogels have shown, modifications to matrix characteristics can affect angiogenesis, regenerative responses, and macrophage phenotype.^88^ These findings suggest that cross-linking could become a biological design variable instead of a formulation parameter.

#### 6.1.2. Dynamic hydrogels as biophysical switches

The concept that hydrogels are dynamic biophysical switches rather than merely passive chemical carriers is supported by this tactic. The primary function of a passive carrier is to hold and release therapeutic drugs, whereas a biophysical switch can alter the physical environment that cells face in response to mechanical loading, pH, reactive oxygen species, enzymatic activity, redox conditions, or cellular remodeling. Molecular mobility, viscoelasticity, mesh size, rigidity, and network connection can all be altered by the formation, dissociation, or exchange of dynamic crosslinks, which serve as the molecular basis for this behavior.^89^ Because the biochemical and mechanical environments alter during the healing process, this dynamic feature is especially pertinent to chronic lesions. A cohesive hydrogel can preserve hydration, prevent bacterial invasion, protect, and retain therapeutic medications during the inflammatory stage. When inflammation declines, the network's controlled relaxation and disintegration can encourage the migration of fibroblasts, endothelial cells, and keratinocytes, improving cellular accessibility. Progressive network reconfiguration can promote extracellular matrix formation and deposition during tissue remodeling.^90^ Future systems should therefore be designed to give a variety of mechanically and structurally diverse states in accordance with the shifting biological requirements of healing, rather than creating a hydrogel with a single constant mechanical state. As a result, smart wound dressings that provide both mechanical protection and controlled release are increasingly utilizing such dual-network systems. A comparison of hydrogels used in wound healing cross-linking techniques is shown in Table 4.

**Table 4:** Comparison of crosslinking strategies for wound healing hydrogels

Crosslinking type	Molecular mechanism	Mechanical/viscoelastic behavior	Advantages	Limitations	Wound-healing relevance	Ref.
Physical non-covalent	Hydrogen bonding, ionic interactions, hydrophobic association, host–guest interactions	Rapidly reversible bonds; efficient energy dissipation and stress relaxation; often strong shear-thinning/self-healing but lower long-term structural stability	Mild processing, injectable, reversible, cell-compatible	Relatively weak mechanical stability; interactions can be disrupted by salts, dilution or physiological conditions	Injectable and temporary matrices; rapid adaptation to mechanical deformation	91
Chemical- covalent	Stable covalent bonds formed by photo-crosslinking, click chemistry, enzymatic or chemical reactions	Low bond exchange; relatively stable modulus and limited stress relaxation unless network degradation occurs	High strength, dimensional stability, controlled degradation	May restrict cell-mediated remodeling; some systems require initiators/crosslinkers	Long-lasting barriers, structural support and controlled release	92
Dynamic covalent	Reversible covalent bonds such as Schiff-base/imine, boronate ester and disulfide exchange	Slower and more chemically defined bond exchange than physical interactions; permits stress relaxation, self-healing and network remodeling while retaining greater cohesion than purely physical networks	Adaptive mechanics, self-healing, stimuli responsiveness and controlled remodeling	Exchange kinetics depend strongly on pH, redox state, competing molecules and network chemistry	Adaptive wound matrices and temporally controlled tissue regeneration	93
Hybrid/dual-network	Permanent covalent network combined with physical or dynamic covalent bonds	Permanent network maintains integrity while reversible bonds dissipate energy and enable stress relaxation; mechanical response can be independently tuned	Combines strength, toughness, self-healing and adaptability	Greater formulation and manufacturing complexity	Advanced smart dressings requiring both mechanical protection and dynamic cellular remodeling	94

### 6.2. Nanocomposite hydrogels, 3D/4D printing approaches, encapsulation of drugs and controlled release

By adding characteristics like improved mechanical performance, electrical conductivity, or antibacterial efficiency, the use of nanoscale fillers has dramatically changed hydrogel design. The most often used nanofillers are metal nanoparticles (Ag, ZnO, CuO, and Au), which are recognized for their broad-spectrum antibacterial properties by rupturing cell membranes and generating reactive oxygen species. However, cytocompatibility and antibacterial qualities must be balanced. Plant or bacterial cellulose is the source of nanocellulose, a sustainable and biocompatible reinforcement that increases tensile strength and swelling stability while permitting the porosity required for gas and nutrition exchange. These hybrid matrices have improved antibacterial and electro-conductive properties thanks to the application of graphene oxide (GO), which promotes angiogenesis and fibroblast development. Similar to the natural extracellular matrix (ECM), graphene oxide's oxygen-containing functional groups increase hydrophilicity and protein binding.^95,96^ Micro- and nano-patterning techniques (such as photolithography, soft lithography, and electrospinning) are used to create hydrogel surfaces that affect cellular alignment, mobility, and specialization.

By mimicking the fibrous structures found in the natural extracellular matrix, these topologies promote keratinocyte organization and enhanced epithelial repair. Concurrently, 3D bioprinting makes it possible to create wound dressings with bioactive materials and spatial gradients of stiffness. For example, the outer layer may contain antibacterial nanoparticles, while the inner layer may have pro-angiogenic growth factors. In order to adapt to uneven wound shapes and enable the scheduled release of medications, 4D printing uses materials that change their shape or function in response to stimuli such as temperature, pH, or moisture.^97^ Hydrogels serves as protective containers for the controlled release of growth hormones, antimicrobial agents, and cells. Diffusion through the polymer matrix, degradation rate, or sensitivity to stimuli (pH, reactive oxygen species, or enzyme activity) all control drug release. Multilayered hydrogel systems often combine an initial fast release of antimicrobials with a prolonged release of regeneration chemicals (like VEGF and bFGF). Similar encapsulation maintains metabolic activity and paracrine communication while protecting living cells, including fibroblasts and stem cells, from oxidative damage and immunological attack. By encouraging the growth of granulation tissue and angiogenesis, this cell-dense method speeds up wound healing.^98^

## 7. Mechanistic insights in wound healing

By preserving a moist healing environment, absorbing extra exudate, and offering a three-dimensional scaffold that promotes cell adhesion and migration, hydrogels interact with wound tissue. Fibroblast proliferation and extracellular matrix deposition are encouraged by integrin-binding sites found in natural polymer hydrogels that comprise collagen, gelatin, or hyaluronic acid. Additionally, hydrogels' viscoelastic qualities influence macrophage polarization from a pro-inflammatory M1 to a pro-healing M2 phenotype, which is essential for reducing chronic inflammation.^99^ Depending on the polymer crosslink density and network hydrophilicity, drug release from hydrogels occurs *via* Fickian diffusion, degradation-controlled, or swelling-controlled mechanisms. ROS- and pH-responsive hydrogels that selectively release medications in contaminated or inflammatory microenvironments are examples of advanced designs. To achieve predictable, localized administration without systemic toxicity, mesh size and degradation rate optimization has been driven by mathematical modelling.^100^

Endothelial invasion and the development of new capillaries are supported by hydrogels that imitate the nanoarchitecture of the extracellular matrix (ECM) by containing peptide motifs (RGD), protease-cleavable linkers, or angiogenic ions (Cu^2+^, Zn^2+^). The regenerating tissue receives oxygen and nutrients more quickly as a result of this vascularization.^101^ Multifunctional hydrogels modulate the immune response while controlling infection through incorporated antimicrobials or nanoparticles. They assist in transitioning the wound from the inflammatory to the proliferative phase by lowering the bacterial burden and ROS levels. By permitting cell infiltration and ECM replenishment at the proper rates, controlled degradation further supports tissue remodeling. Because of these synergistic mechanisms, nanocomposite and stimuli-responsive hydrogels are among the most promising options for wound dressings in the future.^102^ The mechanistic aspects, mechanisms and outcomes are presented in Table 5.

**Table 5:** Mechanistic roles of hydrogels in wound healing

Mechanistic aspect	Key function	Representative mechanism	Biological outcome	Ref.
Cell–matrix interaction	Adhesion, migration	Integrin–ligand binding	Re-epithelialization	99
Controlled drug release	Sustained therapy	ROS/pH response	Localized antimicrobial action	100
Angiogenesis	ECM mimicry	Cu^2+^/Zn^2+^ release, VEGF binding	Capillary formation	101
Infection & inflammation control	ROS scavenging, antimicrobial	NP or peptide release	Reduced bioburden, balanced cytokines	102

### 7.1. Biophysical regulation of cell fate and tissue-interface maturation

A smart hydrogel's regeneration activity is influenced by its three-dimensional network's physical information as well as its chemical makeup and therapeutic payload. Cells at the edge of the wound are constantly sensing topography, ligand density, pore architecture, matrix stiffness, viscoelasticity, mechanical stress from nearby cells, and tissue deformation. These physical cues are transformed into intracellular biochemical reactions that affect cell motility, proliferation, differentiation, inflammatory phenotype, and extracellular matrix formation *via* cell–matrix adhesions and cytoskeletal mechanotransduction. As a result, a hydrogel can serve as more than just a passive drug reservoir; it can also be an instructional microenvironment. Matrix stiffness is crucial as variations in polymer cross-linking and network density influence the mechanical resistance encountered by adhering cells. However, stiffness is not to be seen in isolation from stress relaxation. A matrix distinguished by a fairly high initial modulus and quick stress relaxation can deliver mechanical reinforcement while permitting gradual cellular reorganization, whereas a continually elastic framework might obstruct cell-induced deformation and constrain matrix reconfiguration. Therefore, dynamic covalent connections, reversible physical contacts, enzyme-sensitive linkers, and degradable polymer components can be amalgamated to develop matrices that adjust their mechanical traits in accordance with biological activity or stimuli associated with wounds. This temporal modulation could more properly depict the progressive mechanical conditions faced throughout the change from inflammation to proliferation and tissue remodeling.^103^ Geographical considerations also influence the modulation of biophysical signals. Within a single construct, many microenvironments can be created by variations in stiffness, ligand levels, pore size, fiber orientation, and biological signals. These gradients may facilitate the coordinated migration of keratinocytes, fibroblast infiltration, endothelial cell architecture, and the gradual deposition of new extracellular matrix along the edge of the wound. Additionally, directional signals that influence cell alignment and collective movement may be provided by micro- and nanoscale patterning. Therefore, structured hydrogels and scaffolds should be designed to replicate the biochemical components as well as the mechanical and structural variety of genuine tissue interactions.

Customized ECMs offer a crucial mechanical standard. By showing that an engineered extracellular matrix based on fibrillar fibronectin, supported by a porous polymer scaffold, more effectively promoted neurogenesis, glial maturation, and neuronal diversity than standard matrigel, Muñiz *et al.* illustrated the impact of defined matrix architecture and cell–matrix interactions on cellular development.^104^ This study highlights a broader concept that could be applied to regenerative wound engineering, even if it concentrates on neural tissue: the structure of the matrix could act as a biological guidance instead of merely a physical base. In a similar vein, developmental engineering approaches show how matrix composition, geometric constraints, signaling, and other boundary conditions can influence multicellular organization and tissue formation. According to this theory, a hydrogel must provide mechanical and metabolic cues for wound healing that are temporally coordinated. A cohesive and adhesive matrix can provide protection and maintain localized therapeutic components throughout the early stages of inflammation. Regulated stress reduction and network reconfiguration can improve cellular availability and facilitate fibroblast and endothelial penetration as inflammation decreases. Collagen accumulation, extracellular matrix architecture, vascular growth, and re-epithelialization can all be facilitated by the matrix's changing mechanics and degradability during proliferation and remodeling. Therefore, it is unlikely that the ideal matrix will have a single static mechanical condition; rather, it should provide a range of biophysical states that correspond with the evolving requirements of the regenerating tissue.^105^ As a result, this method modifies the standards for evaluating hydrogel performance. Bulk modulus, swelling, degradation, and total drug release are usually highlighted in traditional characterization. Stress-relaxation kinetics, loss and storage moduli, matrix remodeling, ligand availability, spatial stiffness gradients, cellular traction or spreading, migratory patterns, and tissue-interface layout should all be included to these evaluations for regenerative reasons. These evaluations would allow researchers to determine whether a hydrogel actively controls cellular arrangement or merely maintains structural coherence.

### 7.2. Synergistic coupling of antimicrobial, immunomodulatory and regenerative functions

Multifunctional hydrogels' therapeutic potential is dependent on both their biological coordination and the existence of numerous functionalities. In chronic wounds, infection, inflammation, and poor regeneration create a pathogenic circle that reinforces itself. Bacterial colonization and biofilm formation stimulate inflammatory signaling and ROS production, whereas excessive inflammation and oxidative stress impair fibroblast function, angiogenesis and extracellular-matrix remodeling.^106^ A rational multifunctional hydrogel should therefore interrupt this pathological loop at several points. The first therapeutic step is reduction of microbial burden. Antimicrobial agents, cationic polymers, metal nanoparticles, antimicrobial peptides or photothermal/photodynamic components can reduce bacterial viability and biofilm formation. However, when ROS-generating or metal-ion-based methods are employed, excessive antimicrobial activity may harm host cells. Therefore, cytocompatibility and antibacterial potency need to be assessed jointly. Resolving abnormal inflammation is the second phase. ROS scavenging, anti-inflammatory compounds, immunomodulatory ions, or substances that affect macrophage phenotype can all be used to minimize excessive inflammatory signaling once the microbial burden is decreased. The shift from inflammation to proliferation may be aided by modulation toward a pro-reparative macrophage state. The third stage is regeneration. Endothelial cells, fibroblasts, and keratinocytes find a more favorable environment when there is less inflammation. Vascularization, collagen deposition, and re-epithelialization can then be encouraged by angiogenic agents, ECM-mimetic motifs, bioactive peptides, growth factors, or suitable matrix mechanics.^107^ This series shows that multifunctionality is not only a collection of cumulative characteristics but rather a dynamic therapeutic cascade. For each therapeutic component in the ideal hydrogel, different release rates or spatial compartments might be required. Macrophage polarization may be affected by the application of heat and ROS to destroy germs. Localized and controlled treatment may boost healing by eliminating bacteria, while an over-accumulated ROS or heat can extend inflammation. The VEGF, PDGF, and TGF-β growth factors are secreted by M2 macrophages to drive tissue repair. The induction of M2 polarization at the inappropriate time, however, can hinder the clearance of bacteria and lead to premature fibrosis, underscoring the importance of timing,^108^ thus high ROS levels or strong antimicrobial agents such as silver ions can also impair the regeneration of tissues by damaging keratinocytes and fibroblasts.

## 8. Preclinical and clinical applications

### 8.1. *In vitro* studies: antimicrobial, cytocompatibility, and immunomodulatory assays

Strong *in vitro* bactericidal and antibiofilm action is regularly shown by multifunctional antimicrobial hydrogels against both Gram-positive and Gram-negative microorganisms. Zone-of-inhibition, time–kill curves, planktonic killing (CFU-count/log-reduction), and biofilm eradication (crystal violet, CFU from broken biofilms, or confocal live/dead imaging) are common assays described in recent articles. In order to exhibit broad-spectrum activity, photothermal or photodynamic hydrogels (PDA, AuNPs, Cu-carbon dots, black phosphorus nanosheets) are often combined with ROS generation or metal-ion release. These hydrogels produce rapid bactericidal effects under NIR irradiation, frequently reporting >90–99% kill within minutes under applied irradiation.^109^ According to latest experimental research, biochemically driven substrates like GelMA-based formulations and ECM-mimetic combinations foster excellent viability of cells (>80–95% at 24–72 h), facilitate propagation in scratch assays, and promote keratinocyte and fibroblast propagation as well as growth, particularly if oxidative damage has been buffered or adhering cell markers (RGD motifs, hyaluronic acid) are found.^110^ For instance, improved fibroblast emigration through PI3K/AKT signaling *in vitro* was described by an injectable liposome-encapsulating polypeptide polymer research, suggesting beneficial matrix behavior.^111^ These investigations highlight that substrate biochemistry and stiffness have an essential effect on how cells behave *in situ*, indicating that a combination of metabolic, structural and physiological assessments (such as migration and angiogenic tube formation) could be included in cellular-compatibility criteria for determining regeneration potential.^112^ Macrophage reactions (RAW264.7, THP-1, primary human macrophages), cytokines characterization (TNF-α, IL-6, IL-1β, IL-10, TGF-β), ROS/NO tests, plus biomarker expressions (iNOS/CD86 for M1; CD206/Arg1 for M2) are the main topics in experimental immune modulating research.^113^

Recently developed multipurpose polymer hydrogels exhibit potential in reducing inflammatory release of cytokines, attenuating cellular damage under cultured cells conditions, and shifting the macrophage phenotype into the healing-promoting M2 profile. These effects can be achieved through the absorption of EVs, which are inherent framework indicators, or as sustained release of small molecules/ions (*e.g.*, Mg^2+^, polyphenols).^114^ Immune-modulating material applications that improve IL-10 release alongside CD206 activity with *in vitro* conditions, laser-crosslinked silk-curcumin-acrylamide nanocomposite hydrogels that suppress M1 markers and have antibacterial properties, and pH-responsive GelMA alloys that encourage the polarization of M2 in pro-inflammatory settings are some examples.^115^ Predictive modeling and fabrication of hydrogels from computational to advanced biomaterial designs are presented in Fig. 8.

**Fig. 8:**
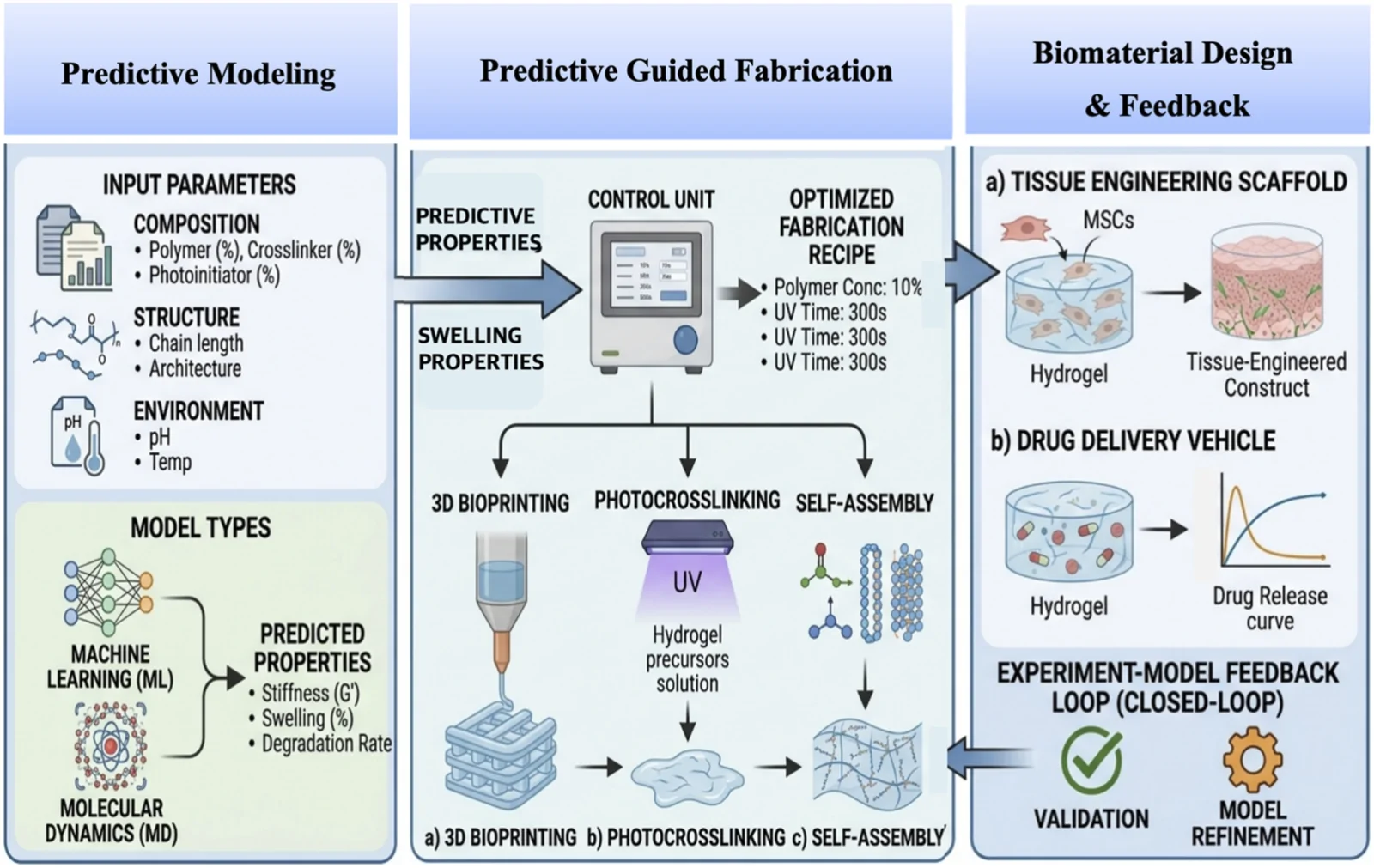
Hydrogel fabrication and predictive modelling for biomaterial design.

### 8.2. *In vivo* studies: animal wound healing models

Multipurpose polymers which combine immune modulation, angiogenic stimulation, as well as antibacterial and ROS-scavenging capabilities have shown markedly faster healing results in a variety of diabetic and infected rodent wound types. For instance, from a previous investigation, ulcers from animals with diabetes were treated utilizing a nano Enzyme activated hydrogel made of GelMA enriched using magnesium ion-based polymeric oxide (Mg-POM). Immunohistochemical findings from the investigations revealed increased CD31^+^ endothelial cell number (showing greater angioplasty) and a shift in macrophage populations toward the M2 phenotype. The hydrogel therapy also boosted cell granulation development as well as the deposition of collagen (as demonstrated through Masson's trichrome).^116^ In a different study, the entire thickness diabetic mouse wounds were nearly entirely sealed in 10–14 days using a self-adjusting hydrogel that is responsive to glucose and oxidative stress (containing metformin, l-arginine, and ascorbic acid liposomes within a gel/PVA matrix). These experiments show that these hydrogels improve vascularization and matrix remodeling, influence the environment of the immune system (less inflammation, increased M2 macrophages), and speed up wound closure (often reducing the wound area by between thirty and fifty percent or more when compared with the control group over a fortnight).^117^ The hydrogel itself substantially outperformed conventional coverings within living cells, achieving greater than 90 percent closing rates through day 14; histology demonstrated improved collagen fiber organization, a greater level of CD31 and α-SMA in granulation tissues, and immunofluorescence revealed elevated CD206 (M2) macrophage marker along with decreased amounts of iNOS protein (M1).^118^ Recently, in diabetes-related wound instances, a moderately photothermal hydrogel containing ZIF-8 nanoparticles as well as intended to disrupt MRSA energy utilization reached roughly 95 percent wound healing in just fourteen days. The healed skin's elastic properties enhanced, the level of collagen I/III ratio increased, along with the localized levels of bacteria as well as reduction in the level of inflammatory cytokines.^119^ Wound healing processes *in vivo* and *ex vivo* models are shown in Fig. 9.

**Fig. 9:**
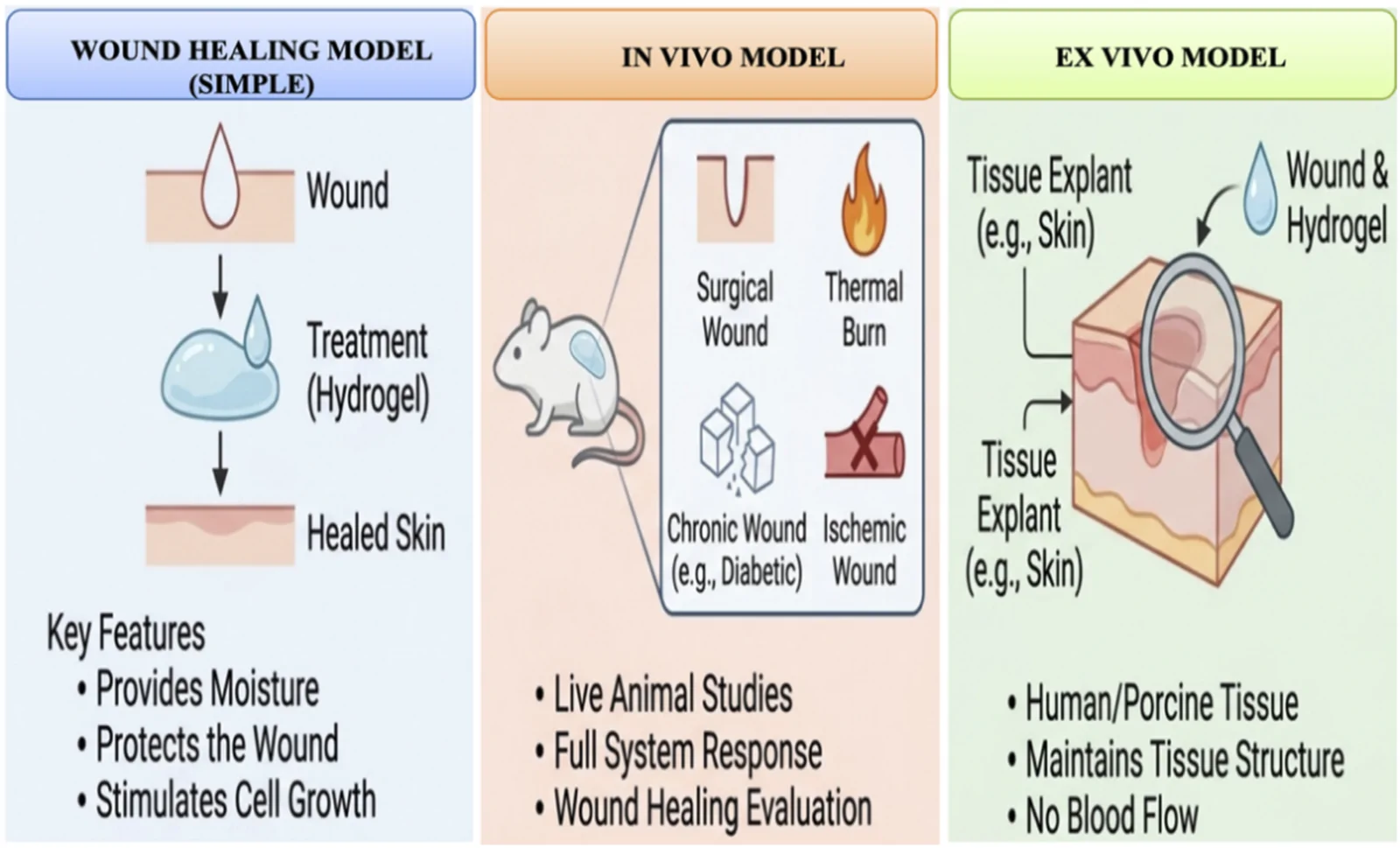
Schematic overview of hydrogel for wound healing in simple, *in vivo* and *ex vivo* models.

#### 8.2.1. Translational limitations of rodent wound models

Rodent wound models remain indispensable for screening hydrogel efficacy because they are relatively inexpensive, genetically tractable and suitable for mechanistic studies. However, their physiology for healing wounds is very different from that of human skin. Human wounds depend more heavily on re-epithelialization and granulation tissue production, but rodent skin has a panniculus carnosus that promotes quick wound contraction. This difference creates an important interpretive problem. A hydrogel may produce rapid reduction in wound area in mice partly because it accelerates contraction rather than because it restores human-like epithelial architecture. Therefore, wound area percentage alone should not be considered a sufficient endpoint for translational evaluation.^120^ Future hydrogel studies should combine wound-area measurements with quantitative assessment of re-epithelialization, granulation tissue thickness, collagen organization, vascular density, inflammatory-cell populations, macrophage phenotype and scar quality. Splinted rodent models can reduce contraction and better approximate human-like closure. For diabetic wounds, models incorporating diabetes together with ischemia, infection or biofilm formation may provide greater clinical relevance than healthy acute excisional wounds. Larger animals, particularly porcine models, can provide additional information because their skin architecture and healing characteristics more closely approximate human skin.^121^ Therefore, small animals should be used for mechanistic screening in a logical translational pathway, followed by more clinically comparable models prior to human trials. Table 6 lists the mechanisms, translational interpretation, and *in vivo* and *in vitro* uses of hydrogels.

**Table 6:** Hydrogels systems, composition, mechanisms, *in vivo*/*in vitro* models, outcomes and translational interpretation

System/category	Representative composition	Principal mechanism	Model/evidence	Main outcome	Translational interpretation
Natural/polymer hydrogel	Chitosan/alginate/HA-based systems	Moisture management, bioactivity, drug delivery	*In vitro*/preclinical	Improved cell compatibility and healing	Relatively established material platforms^122^
Nanocomposite hydrogel	Metal/metal-oxide nanoparticle-containing networks	Antibacterial activity, ROS modulation	*In vitro*/*in vivo*	Reduced bacterial burden and enhanced healing	Requires long-term nanoparticle safety evaluation^123^
Stimuli-responsive hydrogel	pH/ROS/enzyme-responsive network	On-demand release	Diabetic/infected wound models	Localized therapeutic delivery	Promising but predominantly preclinical^124^
3D-printed hydrogel	Borophene/ZnO-containing printed hydrogel	Antibacterial activity + controlled release	Experimental	Spatially defined hydrogel architecture	Supports personalized manufacturing but requires scale-up validation^125^
Magneto-responsive hydrogel	Iron-oxide nanoparticle-containing network	Magnetic control of rheology/release	*In vitro*/material studies	Remote control and tunable release	Potential for advanced controlled delivery^126^
Cell-/EV-containing hydrogel	Hydrogel + cells/exosomes/conditioned medium	Paracrine and regenerative signaling	Preclinical	Enhanced regeneration	Manufacturing and regulatory complexity remain major barriers^127^
Conventional clinical hydrogel dressing	Commercial hydrogel dressing	Hydration and wound-bed management	Clinical studies	Improved wound healing in selected indications	More clinically mature than multifunctional smart systems^128^
Amniotic/biological hydrogel systems	Amniotic-derived components	Anti-inflammatory/regenerative effects	Clinical/preclinical	Improved wound characteristics	Requires biological-source standardization^129^
AI/sensor-integrated hydrogel	Conductive hydrogel + sensors/ML	Monitoring and prediction	Emerging research	Wound-state classification/prediction	Early-stage; clinical validation required^130^

### 8.3. Case studies of hydrogel dressings in clinical trials

In surgical incisions, diabetic ulcers, and other difficult wounds, hydrogel-derived and biologically based wound dressings have demonstrated notable clinical success. Because they provide a physiologically active extracellular matrix-like milieu with structural and signaling components that may encourage epithelialization, lower inflammation, and influence tissue repair, amniotic membrane materials are especially significant. These effects extend beyond passive wound protection since biologically produced matrices can affect cell–matrix interactions and the surrounding inflammatory environment. An amniotic fluid-derived gel formulation was compared to traditional treatment in a triple-blind clinical trial of individuals with diabetic foot ulcers. This study supported the potential clinical value of biologically generated hydrogel formulations in the management of diabetic wounds by reporting improvements in wound characteristics and healing outcomes over the course of the 8-week treatment period.^131^ However, such clinical evidence should not be applied to all multifunctional smart hydrogels; rather, it should be evaluated in accordance with the particular formulation and indication. As regenerative platforms, hydrogel dressings with cell-conditioned media have also been studied. Hydrogel-based administration of cell-conditioned substances has been shown to promote wound repair and modulate inflammatory responses, according to a systematic review.^132^ Additionally, the identical analysis highlights the importance of periodic disclosure of immunological biomarkers in studies involving such coverings. The analysis highlights the component's intrinsic anti-bacterial, anti-inflammatory, anti-fibrotic, and stimulating epithelialization properties in embryonic barrier usage. All things considered, such data sets indicate that hydrogel dressings can actively produce a cell-compatible as well as immunoregulatory wound milieu in addition to being autonomous moisture-holding applications.^133^ In another specialized research investigation, a proficient scaffold configuration incorporating glial and stem cells, with delivery mechanisms for neurotrophic factor and cytokine dissemination, enhances regenerative capacity. A fascinating area of study that gives conduit design another dynamic component is bioelectrical interactions. Better patient outcomes and clinically significant outcomes are obtained by progressively using these strategies.^134^ These findings show that hydrogel dressings can offer more than simply physical protection and moisture preservation; well-designed systems may produce immunoregulatory and cell-compatible wound microenvironments. Differentiating between the early translational status of very complex stimuli-responsive, nanocomposite, or AI-integrated hydrogel systems and the clinical maturity of traditional or biologically generated hydrogel dressings is critical.

## 9. Translational challenges

Despite encouraging laboratory results for cellular compatibility and immune suppression, a number of important material and biological limitations continue to impede the translation of multifunctional hydrogels into therapeutic wound care solutions. For instance, numerous hydrogels are designed to promote adhesion of cells and expansion *in vitro* and deliver immunomodulatory cues (like cytokine release or macrophage polarization), but little is known about their long-term biocompatibility, degradation behavior, and potential nanomaterial accumulation. According to one analysis, there is still a lack of comprehensive assessment of hydrogel degradation by-products, potential nanomaterial accumulation toxicity, and immunomodulatory effects particularly regarding chronic tissue responses immediately following implantation.^135^ Silver nanoparticles and ROS/photothermal systems have a narrow therapeutic window when it comes to antimicrobial failures. Some systems might fail to treat irregular wounds, and doses required to kill biofilms may also cause harm to keratinocytes and neighboring tissues. *In vitro* polarization of M1 to M2 may be achieved with the use of EV and cytokine loaded hydrogels, but *in vivo* it may be of short duration due to the rapid burst of the loaded cytokines, as these cytokines are required for long-term immune remodeling during chronic wound healing.^136^ There are also situations where growth-factor- and stem-cell-based hydrogels have performed well in the rodent model that do not perform well in human trials, emphasizing the fact that results for animals do not necessarily translate to humans. Additionally, while cytocompatibility studies (*e.g.*, cellular survival and keratin cell migration) were useful, they often fail to capture the complex injury microenvironment (bacterial infection, oedema, and continuous aggravation) that the hydrogel must operate in. Flexibility is another challenge: when organic polymer-derived hydro gels are transferred from the testing bench to the incision region, they may exhibit breaking and generation-to-generation variation, making production-wide licensing even more challenging.^137^ Despite their promising *in vitro* cytocompatibility and immunomodulatory qualities, multifunctional smart hydrogels are difficult to translate clinically. One major disadvantage is regulatory classification: these hydrogels often come under the category of, “combination products,” requiring a thorough evaluation to determine if they belong in the category of innovative combination products, medical equipment, or medicinal items. The bioactive or medicinal component's nature (well-characterized or unique), the hydrogel's overall intended therapeutic action, the urgency of the patient's needs, and the duration and method of the hydrogel's numerous actions are all factors that affect this classification.^138^ Although clinical studies support the general utility of hydrogel-based wound dressings, these findings should not be interpreted as evidence that all multifunctional smart hydrogels are clinically effective. Conventional hydrogel dressings and next-generation stimuli-responsive nanocomposite systems differ substantially in composition, manufacturing complexity, mechanism and regulatory status. Thus, evidence from conventional hydrogel dressings provides clinical support for the hydrogel platform but does not establish clinical efficacy for experimental multifunctional formulations.

## 10. Limitations and future perspectives of hydrogels in wound healing

The development of multifunction smart hydrogels to aid in the healing of wounds has advanced remarkably, but their widespread clinical application is hampered by a number of important issues. Scalability is one among the main issues; numerous hydrogel compositions depend on intricate manufacturing techniques, including numerous stages of crosslinking or the addition of therapeutic nanotechnologies that are challenging to reliably replicate on a large-scale.^139^ Widespread usage in medicine is severely hampered by the cost of production and raw ingredients, especially for hydrogels that contain pricey functional chemicals, growth accelerators, or sensitive nanoparticles. For innovative hydro gels to advance toward beneficial uses in chronic wound healing, these challenges must be addressed by standard manufacturing procedures, enhanced sterilizing techniques, and economical production processes.^140^ Multifunctionality by itself does not ensure better treatment performance, despite significant advancements, as evidenced by a number of persistent issues. First, while a higher concentration of nanoparticles may improve antibacterial action, it may also increase oxidative stress, cytotoxicity, or long-term tissue retention. Second, strongly crosslinked networks impede therapeutic diffusion and limit cellular migration while offering exceptional mechanical strength. On the other hand, loosely crosslinked systems could offer quick release but don't have enough structural integrity.^141^

The use of short-duration acute wound models to bolster claims about chronic wounds is another prevalent drawback. Simple acute excisional wounds cannot adequately replicate the extended inflammation, altered vascularization, metabolic dysfunction, biofilm formation, and compromised immune responses associated with chronic diabetic and infected wounds. Incomplete reporting of degradation products, long-term material retention, sterilization effects, and batch-to-batch repeatability are further problems in the sector. When formulations include several polymers, nanoparticles, medications, peptides, cells, or biological elements, these problems become more significant.^142^ A further concern is overengineering. Combining many therapeutic components can increase manufacturing complexity and regulatory burden without necessarily producing synergistic biological effects. Therefore, future systems should demonstrate the contribution of each major component and, where possible, compare multifunctional formulations against appropriate single-function controls. The most clinically relevant design principle is consequently not maximum functionality but optimized functionality with demonstrable therapeutic necessity, safety, manufacturability and translational benefit.^143^

## 11. Integration of artificial intelligence smart systems in hydrogels

Artificial intelligence (AI) is emerging as a complementary technology for the design, fabrication and monitoring of smart hydrogel systems. Reducing the number of formulation iterations needed to find materials with predetermined mechanical, rheological, transport, or biological qualities is its biggest immediate benefit rather than replacing experimental validation.^144^ Hydrogel-based medicines' accessibility and affordability for patients and healthcare institutions will depend on striking a balance between cost and innovation, which calls for collaboration between researchers, physicians, businesspeople, and legislators. Because of their special and versatile qualities, hydrogels have the potential to significantly enhance patient outcomes, lower healthcare costs, and advance the delivery of care for long-term diabetes-associated wounds, according to the expanding body of research.^145^ Hydrogel-based rehabilitation solutions must be incorporated into clinical practice using patient-centered, pragmatic techniques in conjunction with continuous technological improvements in order to fully achieve this promise.

### 11.1. Artificial intelligence and machine learning for smart hydrogel design, fabrication and wound monitoring

Advanced hydrogel systems may now be rationally designed, optimized, fabricated, and monitored with the help of artificial intelligence (AI), especially machine learning (ML). Conventional hydrogel development generally relies on iterative experimentation in which polymer composition, concentration, crosslinking conditions, nanoparticle loading, rheological properties and processing parameters are independently optimized. This approach becomes increasingly inefficient as the number of formulation variables increases. A recent review specifically highlighted the growing integration of AI with hydrogels for material design, performance optimization, 3D printing, biosensing and wound-healing applications. AI-based approaches can complement experimental studies by identifying nonlinear relationships between formulation variables and material properties, narrowing the experimental design space, predicting fabrication outcomes and enabling data-driven optimization.^146^ Currently, the use of AI/ML in multifunctional wound hydrogels is still in its early stages of development and research is divided into three areas: One, predictive material design using ML/QSAR can predict a material's properties before the need for actual synthesis, such as the crosslinking density, swelling, and drug release, and is mostly at the computational or proof-of-concept level (TRL 2–3). Second, AI/parameter-optimized fabrication that ties the digitally controlled 3D-printing parameters to the properties of the hydrogel, which include antibacterial activity and drug release, is a more advanced proof-of-concept (TRL 3–4).^147^ Third, a new paradigm of sensing-and-modulating systems that are closed-loop, in which a physiological signal is collected and a treatment is delivered externally when the system is triggered by the signal, is a close parallel to future AI-based wound care systems (TRL 3–4), but current applications are mostly laboratory validation.^148^ Most importantly, it is yet a system that does not show a completely autonomous ML decision layer that receives the real-time wound-sensor data and automatically regulates hydrogel therapy. So, AI-powered wound hydrogels should be viewed as a new, rather than existing, clinical technology, but instead as an emerging combination of predictive design, digitally controlled fabrication, and physiological sensing. For chronic wound management, the potential role of AI extends beyond formulation optimization. Smart hydrogels increasingly incorporate multiple functions, including antimicrobial activity, controlled drug delivery, conductivity, environmental sensing and tissue regeneration. Consequently, AI can potentially connect material composition, network properties, therapeutic response, wound-state information, providing a framework for more adaptive and personalized hydrogel systems.^149^ However, most current applications remain at the materials-development or prototype stage, and substantial challenges related to dataset quality, external validation, interpretability, reproducibility and clinical translation remain.

### 11.2. AI/ML assisted hydrogel formulation and materials-property prediction

The properties of a hydrogel are governed by multiple interacting variables, including polymer type and molecular weight, polymer concentration, crosslinking density, functional-group concentration, nanoparticle content, solvent composition, pH, ionic strength and processing conditions. These variables can influence swelling, degradation, elasticity, viscoelasticity, mesh size, diffusivity and therapeutic release simultaneously. Traditional one-factor-at-a-time optimization is therefore poorly suited to highly multivariable hydrogel systems. ML provides an alternative approach in which experimentally measured formulation and characterization data are used to construct predictive relationships between input variables and target properties. Algorithms including random forest (RF), support vector machines, decision trees, artificial neural networks, and deep learning models can be used, depending on the dataset and goal. Before conducting comprehensive experimental testing, these models may be able to discover formulation regions linked to desired mechanical strength, swelling behaviour, degradation rate, or release kinetics.^150^ The capacity of AI/ML to capture nonlinear relationships that could be challenging to characterize using traditional regression is a significant advantage. However, the quantity, caliber, and diversity of the training dataset have a significant impact on prediction performance. Models developed using a narrow range of polymer chemistries may perform well within the original experimental space but fail when extrapolated to chemically distinct hydrogel systems. Recent reviews have emphasized that AI-assisted hydrogel research is moving from empirical optimization toward data-driven material-property prediction, while also highlighting limitations associated with data availability, model interpretability and generalizability,^151,152^ therefore, AI should currently be regarded as a decision-support and experimental-design tool, rather than a replacement for experimental characterization.

### 11.3. AI/ML for hydrogel printability and 3D/4D fabrication

One of the most experimentally demonstrated applications of AI/ML in hydrogel research is prediction of printability. In extrusion-based 3D printing, successful deposition depends on a complex combination of viscosity, shear-thinning behaviour, yield stress, elasticity, recovery kinetics, nozzle diameter, extrusion pressure and printing speed. An ink may have excellent biological properties but remain unsuitable for printing because of poor filament formation, excessive spreading, nozzle blockage or inadequate structural recovery after extrusion. A particularly relevant study evaluated 210 biomaterial formulations comprising 16 different bioactive and smart materials and four solvents using direct ink writing. Decision-tree, RF and DL algorithms were trained to predict whether formulations would produce printable structures. All three approaches achieved greater than 80% accuracy, while the RF model achieved the highest reported accuracy of 88.1%, with a precision of 90.6% and *F*_1_ score of 87.0%. The DL model achieved the highest recall of 87.3% and generated a more granular printability map. Importantly, the resulting models were used to define predicted printability windows, demonstrating how ML can reduce experimental trial-and-error during biomaterial ink development.^153,154^ This approach is particularly relevant to smart wound hydrogels because many advanced systems are being developed for extrusion-based 3D or 4D fabrication. AI-assisted printability prediction could allow researchers to screen combinations of polymer concentration, crosslinker concentration, nanoparticle loading and rheological modifiers before printing. This may be particularly valuable for patient-specific wound dressings in which the geometry and mechanical properties of the construct need to be tailored to an individual wound. Importantly, printability should not be treated as a single material property. It emerges from the interaction between formulation rheology and processing conditions. Therefore, future AI models should incorporate not only composition but also nozzle diameter, extrusion pressure, printing speed, temperature, layer height and post-printing crosslinking conditions.

### 11.4. Interpretable machine learning and rheological descriptors

The relationship between hydrogel rheology and printability has also been investigated using interpretable ML. Rather than treating the algorithm as a black box, interpretable approaches can identify which rheological parameters contribute most strongly to successful filament formation.^155^ A study based on an extensive dataset containing 180 hydrogel formulations and rheological/printability measurements used interpretable ML to identify 13 critical rheological measures associated with printability. The study showed that printability cannot necessarily be reduced to a single universal rheological threshold; instead, multiple rheological characteristics act collectively. The findings provided a physically interpretable framework linking hydrogel flow behavior and elastic properties to filament formation.^156^ This finding has important implications for smart wound hydrogel development. Highly bioactive materials may have complex rheological behavior because of nanoparticles, peptides, cells, extracellular vesicles or dynamic crosslinking interactions. Consequently, an interpretable model that identifies the relative contribution of yield stress, viscosity, elasticity and recovery behavior could be more useful than a purely predictive black-box model.^157^ The combination of rheological characterization and interpretable ML may therefore support a rheology printability biology framework, in which formulation variables are linked not only to printing fidelity but also to cellular viability, degradation and therapeutic function.

### 11.5. Current limitations and translational challenges of AI-enabled hydrogels

AI integration does not inevitably enhance hydrogel performance, despite its significant promise. Translation is now restricted by a number of factors. First, dataset quality and size continue to be significant issues. Due to the length of time required for formulation and biological tests, many hydrogel studies provide comparatively tiny datasets. When complicated deep-learning models are employed, small datasets may lead to overfitting and unreliable predictions. Second, data integration is restricted by the variability of experimental techniques. Combining datasets can be challenging because to variations in polymer molecular weight, cross-linking chemistry, rheological measurement techniques, printing equipment, wound models, and biological outcomes. Third, there is still uncertainty over the generalizability of the model. It's possible that a model developed with one polymer family won't be able to correctly forecast another polymer system. In a similar vein, a printability model created with a particular printer and nozzle shape might not translate to another production system. Fourth, interpretability is especially crucial for applications in the biomedical field. An interpretable model that connects formulation factors to quantifiable physical parameters may be more helpful than a model that forecasts a good formulation without offering mechanistic insight. Fifth, AI predictions require experimental validation. A high classification accuracy within a training dataset does not demonstrate clinical usefulness. Independent datasets, external validation and prospective testing are required before models can be incorporated into clinical decision-making. Finally, sensor-integrated AI hydrogels raise additional issues, including sensor drift, biofouling, signal instability, wireless-data security, patient-specific variability and regulatory classification.^158^ The current literature therefore supports AI as a powerful emerging tool for hydrogel research, but fully autonomous AI-controlled therapeutic hydrogels remain an early-stage concept.

### 11.6. Future and clinical perspective of AI smart hydrogels

The convergence of AI, smart polymer chemistry, biosensing and additive manufacturing could ultimately enable patient-specific hydrogel systems. In such a scenario, wound images and physiological measurements could be combined with material-property databases to identify an appropriate hydrogel composition, predict its mechanical and release behaviour, and optimize its fabrication parameters. A future workflow could involve, patient/wound data, digital wound characterization, AI-assisted formulation selection, predicted rheology and printability, personalized 3D/4D fabrication, sensor-enabled monitoring, AI-based wound-state prediction, adaptive therapeutic response.^159,160^ Such an approach would represent a transition from generalized wound dressings toward data-driven, adaptive and personalized wound management. However, coordinated developments in polymer chemistry, biomedical engineering, artificial intelligence, clinical wound assessment, and regulatory science are needed to accomplish this goal. It will be crucial to have interoperable sensing devices, clinically approved outcome assessments, clear reporting, explainable algorithms, and standardized datasets. Thus, creating strong connections between material descriptors, measured hydrogel properties, biological pathways, and therapeutically significant results should take precedence over improving AI complexity. In general, AI in smart hydrogel research should now be seen as an enabling technology rather than a stand-alone therapeutic mechanism. Formulation screening, property prediction, printability optimization, and sensor-derived wound data interpretation are among its most promising short-term uses. Rheology–printability modeling employing 150 printed hydrogels, interpretable analysis of 180 hydrogel formulations, and prediction models based on 210 biomaterial formulations provide as proof for these applications.^161^ The next crucial step is to include these capabilities into verified workflows where AI predictions result in hydrogel performance enhancements that can be replicated in experiments and, eventually, benefits for wound healing that are clinically significant. Claims regarding autonomous or customized AI-controlled hydrogel therapy should be regarded cautiously until such information is available.

## 12. Hydrogels potential for commercialization in clinical settings, future directions, and regulatory issues

The prospect of accurate, personalized wound care is both an exciting opportunity and a challenging challenge as flexible smart hydrogels get closer to becoming a reality. In the future, gel coatings that control inflammation and adaptive immunity will need to be customized to each individual's distinct healing traits.^162^ For example, they should be tailored to the patient's specific allergen sensitivity, relevant comorbidities (such peripheral vascular disease and metabolic syndrome), and wound microenvironment features (like acidity, ROS levels, and bacterial burden). By combining biological sensing, predictive analytics, and smart hydrogels, polymer treatment offers the ability to track the healing process in real time and provide ongoing correction. For example, hydrogels may be able to release growth factors, antibodies, or other therapeutic compounds on demand if sensors or stimuli-responsive modules are added. Precision treatment may be further made possible by combining patient-derived cells or cell components with advanced manufacturing methods, such as 3D-printed hydrogel dressings based on the geometry of a particular wound.^163^ The long-term durability of smart responsive modules, robust personal-data frameworks (*e.g.*, linking wound imaging to customized hydrogel formulations), and navigating the regulatory pathways for such tailored combination products will all be necessary to overcome these challenges and achieve this goal.^164^ There are still significant legal and commercial barriers to the therapeutic application of multifunctional smart hydrogels, which include antibacterial, anti-inflammatory, and regenerative properties. Many of these systems must go through difficult approval procedures since they belong to the unclear regulatory category of combination products (device + biologic or device + medication). The duration and method of action of the gel, the identification and characterization of the therapeutic or biologic component, and the evaluation of the dangers related to novel compounds are all necessary for these procedures. In order to meet functional and biological suitability requirements, for example, as well as high cleanliness standards, repeatable production, and long-term biocompatibility, recent studies emphasize the need for improved hydrogels for chronic wound treatment.^165^ Hydrogels with sensor or antibacterial properties are starting to appear on the market. However, there is less opportunity for cost-effective scale-up due to the increased complexity of immunomodulatory functions (including cytokine release and macrophage polarization) and personalization, which raises manufacturing costs and regulatory load. For instance, published research on wound-healing hydrogels indicates that stimuli-responsive dressings have potential for environmentally friendly production; nonetheless, sterilization compatibility and the absence of extensive validation testing continue to be major obstacles. Future commercial success of new biomaterials is likely to depend on early alignment of material development with regulatory requirements (*e.g.*, classification as a biologic *versus* a device), strong clinical evidence of an advantage over standard treatment, robust manufacturing innovation, and business models that take cost-effective production and reimbursement into account.

## 13. Conclusion

In conclusion, the development of hydrogel-based wound dressings has transformed tissue restoration by combining the advantages of controlled medication release, bioactive molecule dispersion, and a moist healing environment. Researchers have been able to modify the mechanical strength, rate of degradation, and stimuli-responsiveness of hydrogel matrices by employing state-of-the-art cross-linking techniques such as physical, chemical, and hybrid approaches. Self-healing, flexible, and reversible properties that resemble the natural extracellular matrix (ECM) are further made possible by dynamic bonding, which includes Schiff base linkages, disulfide bonds, and host–guest interactions. Additionally, the antibacterial efficiency, structural integrity, and bioactivity of nanocomposite hydrogels containing metal nanoparticles (Ag, ZnO, Cu), nanocellulose, graphene oxide, or clay nanostructures have been greatly enhanced. The linking of polymer networks with nanoscale fillers improves physical performance and allows for adjustable release kinetics of encapsulated medicines. Angiogenesis, collagen deposition, and quick re-epithelialization all crucial processes in wound closure are further encouraged by the inclusion of bioactive cargoes such growth factors, stem cells, and peptides. Nevertheless, there are still a lot of difficulties in spite of the outstanding advancements. Many hydrogel systems struggle to increase production while maintaining repeatability, have unpredictable degradation kinetics, and have poor long-term stability. The biocompatibility and cytotoxicity of nanoparticles within hydrogels require extensive *in vivo* validation to ensure safety for clinical translation. While chronic and diabetic wounds require prolonged therapeutic activity and persistent antimicrobial defense, current research mostly concentrates on short-term wound models. In order to facilitate on-demand and site-specific drug release, future hydrogels should be engineered to react to various physiological stimuli, such as pH, temperature, reactive oxygen species (ROS), or enzyme activity. Advanced fabrication methods such as microfluidics and 3D bioprinting can be used to spatially pattern cells, nanoparticles, and bioactive for wound dressings tailored to individual patients. By incorporating wearable sensors into hydrogel matrices, it may be possible to monitor wound conditions in real time, such as wetness, pH, or infection status, which could direct customized treatment. Future smart hydrogels should therefore move from multifunctionality by component accumulation toward functionality by biological coordination. The next generation of systems should be designed around measurable wound states and should provide temporally and spatially appropriate antimicrobial, immunomodulatory and regenerative responses. Integration with AI-assisted formulation design, 3D/4D printing and sensor-based monitoring may further enable patient-specific wound management. However, successful translation will require standardized characterization, clinically representative animal models, long-term safety assessment, scalable manufacturing and prospective clinical validation.

## Author contributions

Muhammad Bilal Habib, Laiba Arshad and Muhammad Kamran contributed to the conceptualization, investigation, data curation, formal analysis, and writing of the original draft. Ghanwa Batool, Ameera Shahid contributed to the formal analysis, and writing of the original draft. Taseer Muhammad and Noreen Sher Akbar, carried out data curation, formal analysis, and visualization. Anwarul Hasan contributed to supervision, data curation, validation, and editing of the manuscript. All authors discussed the results and approved the final manuscript.

## Conflicts of interest

The authors declare no conflict of interest.

## Data Availability

This review does not feature any software, or code, nor does it produce any new data.
